# Common gene expression strategies revealed by genome-wide analysis in yeast

**DOI:** 10.1186/gb-2007-8-10-r222

**Published:** 2007-10-19

**Authors:** José García-Martínez, Fernando González-Candelas, José E Pérez-Ortín

**Affiliations:** 1 Sección de Chips de DNA-SCSIE, Universitat de València, Dr Moliner 50, E-46100, Burjassot, Spain; 2 Departamento de Bioquímica y Biología Molecular, Universitat de València, Dr Moliner 50, E-46100, Burjassot, Spain; 3 Instituto Cavanilles de Biodiversidad y Biología Evolutiva and Departamento de Genética, Universitat de València, Dr Moliner 50, E-46100, Burjassot, Spain

## Abstract

**Background:**

Gene expression is a two-step synthesis process that ends with the necessary amount of each protein required to perform its function. Since the protein is the final product, the main focus of gene regulation should be centered on it. However, because mRNA is an intermediate step and the amounts of both mRNA and protein are controlled by their synthesis and degradation rates, the desired amount of protein can be achieved following different strategies.

**Results:**

In this paper we present the first comprehensive analysis of the relationships among the six variables that characterize gene expression in a living organism: transcription and translation rates, mRNA and protein amounts, and mRNA and protein stabilities. We have used previously published data from exponentially growing *Saccharomyces cerevisiae *cells. We show that there is a general tendency to harmonize the levels of mRNA and protein by coordinating their synthesis rates and that functionally related genes tend to have similar values for the six variables.

**Conclusion:**

We propose that yeast cells use common expression strategies for genes acting in the same physiological pathways. This trend is more evident for genes coding for large and stable protein complexes, such as ribosomes or the proteasome. Hence, each functional group can be defined by a 'six variable profile' that illustrates the common strategy followed by the genes included in it. Genes encoding subunits of protein complexes show a tendency to have relatively unstable mRNAs and a less balanced profile for mRNA than for protein, suggesting a stronger regulation at the transcriptional level.

## Abstract 2

A comprehensive analysis of six variables characterizing gene expression in yeast, including transcription and translation, mRNA and protein amounts, reveals a general tendency for levels of mRNA and protein to be harmonized, and for functionally related genes to have similar values for these variables.

## Background

The central dogma of molecular biology [1] states that information runs from DNA to protein. In spite of the increasing number of non-protein-coding genes discovered in the past few years, it is still true that a large part of the genetic information follows the central dogma. Therefore, it would be interesting to evaluate the respective contributions and the balance between all the steps in the flow of genetic information from the gene (DNA) to the final product (protein).

Because the ready availability of protein is its final goal, the complex process of gene regulation should be addressed to this aspect. However, given that mRNA is an obligate intermediate step and because the amounts of both mRNA (RA) and protein (PA) are controlled by synthesis and degradation rates, the desired PA can be obtained following different strategies that should take into account the energy costs of each step, the appropriate speed of response to potential changes in the environment [2], the optimal biological noise [3-5] and the possibility of post-transcriptional and/or post-translational regulatory mechanisms [4]. For instance, a given PA can be obtained by maximizing the transcription rate (TR) with a moderate mRNA stability (RS) to obtain a high RA. Ribosomal proteins are an example of this strategy [6]. In other cases, a high RS compensates for a low TR (reviewed in [7]). Sometimes, a low RA can be compensated for by a high TR for each molecule (individual translation rate (TLRi)) or vice-versa [8]. Understanding how PA is related to RA and how RA depends on TR and RS is essential for interpreting the different strategies for gene expression. The stability of the protein molecule (PS) is the final variable determining PA [9]. In general, there is a positive correlation between RA and PA [8,10,11], although it has been shown that in many cases the amount of mRNA is not a good predictor of the amount of protein [12]. The correlation depends critically on the functional categories of genes and proteins [8,13]. Mechanisms for regulating expression at each of these levels have been shown in many organisms, including yeast [7,12,14].

**Figure 1:**
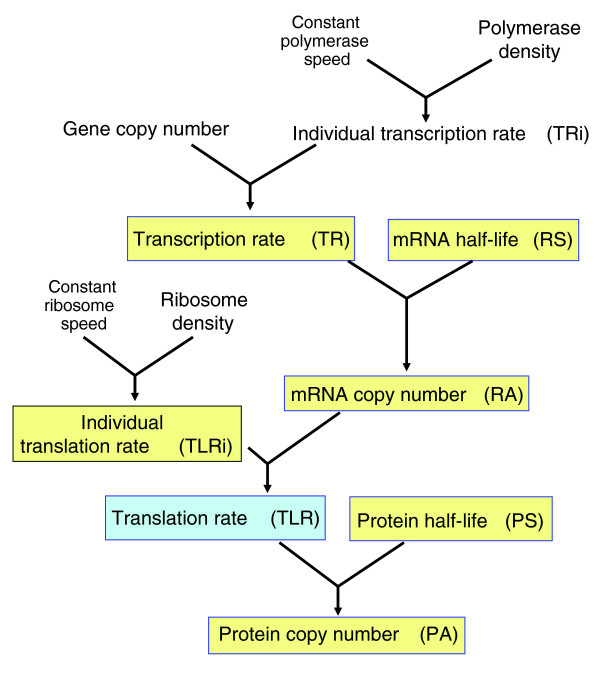
Schematic representation of the steps in the gene expression flow from DNA to protein. Convergent lines with arrowheads indicate the two variables that are combined to generate the next one. In this flow there are two synthesis steps, transcription and translation, yielding mRNA and protein molecules, respectively. The amount of such molecules (RA and PA, respectively) is the consequence of a balance between their synthesis and their degradation. Individual transcription rates (TRi and TLRi) multiplied by copy number gives the total transcription and translation rates (TR and TLR). Whereas synthesis rates are calculated as the number of molecules synthesized in a given time, degradation is expressed here as the half-life of the molecule. The RA depends only on its TR and stability (RS). The PA depends not only on its TLR and stability (PS) but also on the RA. Highlighted in yellow are the variables used in this study that have been obtained experimentally and in blue those that have been mathematically calculated from other studies.

The yeast *Saccharomyces cerevisiae *is probably the most intensively studied organism using functional genomics technologies. In spite of a recent comprehensive study on *Schizosaccharomyces pombe *[15], *S. cerevisiae *remains the only organism for which all the six variables in the genetic expression flow (Figure 1), that is, mRNA amounts [16,17], abundance of many proteins [4,8,11,18], transcription rates [19], translation rates [20,21], mRNA stabilities [19,22,23] and protein stabilities [9], are available. All these data have been obtained independently by different laboratories using standard growth conditions and the same genetic background (S288c). As a consequence, it is now possible to study, for the first time, how a cell regulates the quantities of each of its proteins by adjusting the synthesis rates and stabilities of mRNAs and proteins.

In this paper we analyze the relationships between all six variables under yeast exponential growth in yeast extract-peptone-dextrose (YPD) culture medium. Our analyses show that functionally related genes tend to have similar values for the six variables, which demonstrates that yeast cells use common expression strategies (CESs) for genes in the same physiological pathways. Accordingly, each functional group can be defined by a 'six variable profile' (6VP) that illustrates the strategy followed by that particular group. It is also shown that synthesis rates and molecule amounts tend to be more highly correlated than stabilities. The unique behavior of RS for many genes involved in stable protein complexes suggests that, for those groups, regulation at the transcriptional level is particularly important.

## Results

### Variables acting on the genetic information flow

The recent availability of high-throughput data from the yeast *S. cerevisiae *[8,9,17,20,22,23] opens the possibility of analyzing the relationships between the six variables that control gene expression (TRi, RA, RS, TLRi, PA and PS; Figure 1) at a genome-wide level. In the flow of genetic information, there are two synthesis steps, transcription and translation, which produce (relatively) unstable macromolecules, mRNA and protein. The amount of mRNA depends only on its transcription rate and stability [2,24], while the amount of protein depends not only on its overall translation rate (TLR) and stability but also on the RA [24].

The actual production rates of mRNA and protein, TR and TLR, are, in fact, the product of individual rates, TRi and TLRi, times the number of genes or mRNA copies, respectively. In this case, these two variables are practically equivalent for calculating TR because almost all yeast genes are single copy. Therefore, we have used TR throughout this paper. However, given that TLR and TLRi are essentially different, in this study we have used TLR, TLRi or both, depending on the specific goal of each analysis.

### Correlation between variables

An essential question in molecular biology is to determine which strategy the cells adopt to obtain a given amount of mRNA and protein from each gene and whether the strategies are similar or different for both molecules. Since the amount of each molecule depends on the corresponding synthesis and degradation rates then the use of similar or different strategies for mRNA and protein will affect the correlations between TR and TLRi, and between RS and PS. Moreover, cross-correlations between synthesis rates or stabilities with the amounts of the respective products, mRNA or protein, will inform about the contributions of TR and RS to RA and TLRi and PS to PA.

**Figure 2:**
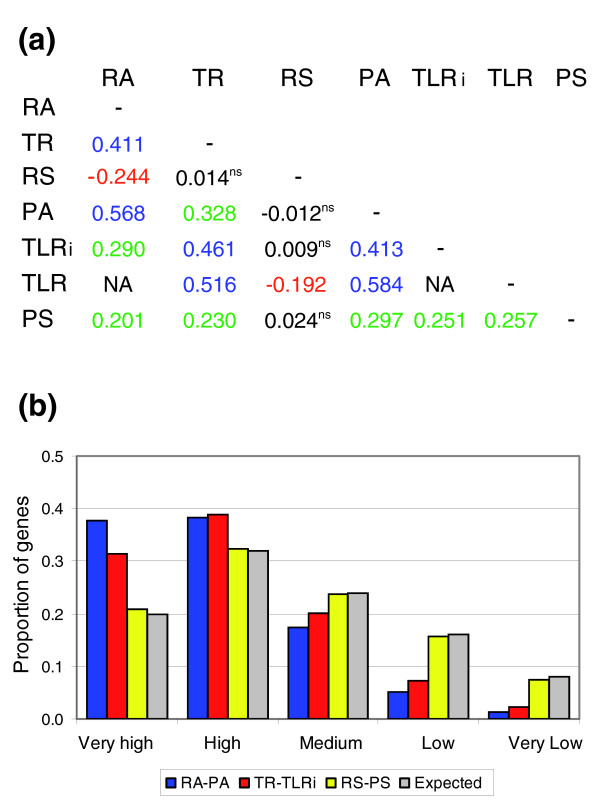
Correlations between variables. **(a) **Spearman rank correlation coefficients for all pair-wise comparisons between the six variables. All the correlations were significant (p < 0.001) except those marked as 'ns'. NA, not applicable. **(b) **Correlations between variables of the same type. Correlations were analyzed by ranking the six variables for all the genes, dividing them into quintiles (1 to 5 from higher to lower values; Additional data file 7) and comparing the positions of the two analyzed variables for each gene. Correlations for genes whose variables were included in the same quintile were considered as 'very high'; if they differed in one unit, they were considered 'high', and so on. A difference of four units was considered a 'very low' correlation. The ordinate indicates the proportion of genes in each correlation category. The expected values (grey) were obtained from a random distribution of all possible quintile combinations.

Pair-wise correlations between the seven variables considered were obtained using Spearman rank coefficients (Figure 2a). We found relatively high, positive, statistically significant correlations (numbers in blue) between RA and PA, PA and TLR or TLRi, RA and TR and between TR and TLR or TLRi. Some of these correlations have been described previously [8,11,17,19]. The correlation between TR and TLR was expected because of the known correlation between TR and RA and the involvement of RA data in the computation of TLR. However, the new, positive correlation (r_S _= +0.46) found between TR and TLRi means that yeast cells tend to use similar synthesis strategies for mRNA and protein. Although this correlation can be influenced by some groups having either high TR and TLRi (ribosome, proteasome) or low TR and TLRi (cell cycle) the relationship is maintained even after eliminating both the 10% higher and lower data points (trimmed r_S _= +0.39). We also found a low positive correlation between PA and TR, RA and TLRi, and PS with all the other variables but RS (numbers in green in Figure 2a). Whereas the PA-TR positive correlation might be explained by the link between TR and RA and the link between RA and PA, the low but statistically significant positive correlations of PS with all the other variables (except, interestingly, RS) is noteworthy. On the contrary, RS tends not to be correlated (numbers in black) or has negative (numbers in red) correlations with the other variables. This is a new finding that will be discussed below.

To better understand the processes underlying the detected correlations, we looked for Gene Ontology (GO) categories enriched in some specific correlations. For this, we first analyzed the correlations between variables of the same type (amounts, individual rates and stabilities) by ranking the corresponding values for the 4,215, 5,590 and 2,618 genes, respectively, for which data on mRNA and protein were available (Additional data files 8 and 13), then divided the list into quintiles (1 to 5 from higher to lower values) and finally compared the positions of the two analyzed variables for each gene. The correlations between the three pair-wise comparisons were classified into five categories ('very high', 0; 'high', 1; 'medium', 2; 'low', 3; or 'very low', 4) by considering the absolute difference between the quintile values for the two variables in each comparison, as described in Materials and methods. As can be seen in Figure 2b, the 'very high' and 'high' correlation categories were over-represented in RA/PA comparisons (Χ^2 ^= 1329.8, df = 4, *p *< 0.0001) and TR/TLRi (Χ^2 ^= 981.7, df = 4, *p *< 0.0001) but not in those between RS and PS (Χ^2 ^= 2.31, df = 4, *p *= 0.677). From these results, it can be concluded that cells coordinate the amounts of mRNA and protein for most genes and that this is achieved mainly through coordination of the synthesis rates, and not of the stabilities, for the two molecules.

After looking for GO categories statistically enriched in the five levels of correlations, we found that some of them were very significant in the 'high correlation' classes, involving high abundance or synthesis rates (quintiles 1-2), most notably cytosolic ribosome, protein biosynthesis, hydrogen transport, redox activity and proteasome, among others (Table 1). Other GO categories were found only in the abundance, but not in the rate, classes (for example, carboxylic acid metabolism, ribosome biogenesis, and so on), or in rate classes only (such as mitochondrial ribosome). There were also GO categories highly represented in the low abundance and/or rate classes (quintiles 4-5): cell cycle, DNA metabolism, DNA binding, regulation of transcription, response to stimulus, and so on. Many of them were related to regulation or control processes. The general trend is that amounts of mRNA and protein are correlated mainly by coordinating their synthesis rates, either if they correspond to abundant proteins, such as the ones belonging to macromolecular complexes, or to scarce ones, such as those involved in regulation.

**Table 1:** Gene Ontology categories over-represented in some comparisons between variables

	Rates				Amounts			
		
	GO*	P^†^	a-P^‡^	No. of genes^§^	GO	P	a-P	No. of genes
**High correlation**								
Both low level (4-5)	Cell cycle	<E-17	<0.001	159/370	Cell cycle	<E-7	0.001	112/300
	Meiosis	<E -6	0.003	52/122	-			
	Regulation of physiological process	<E-16	<0.001	204/518	Regulation of physiological process	<E-10	<0.001	171/459
	DNA binding	<E -12	<0.001	88/190	DNA binding	<E -8	<0.001	66/146
	Protein kinase activity	<E -13	<0.001	64/117	-			
	DNA metabolism	<E -11	<0.001	158/422	DNA metabolism	<E -5	0.017	39/372
	Response to endogenous stimulus	<E -6	0.004	65/164	-			
	Regulation of transcription	<E -10	<0.001	120/298	Regulation of transcription	<E -6	0.001	99/263
	-				RNA splicing	<E -6	0.002	47/103
	Lipid kinase activity	<E -5	0.005	8/8	-			
Both high level (1-2)	Cytosolic ribosome	<E -24	<0.001	93/147	Cytosolic ribosome	<E -78	<0.001	149/156
	Protein biosynthesis	<E -15	<0.001	179/439	Protein biosynthesis	<E -48	<0.001	247/417
	Hydrogen ion transporter activity	<E -6	0.001	25/43	Hydrogen ion transporter activity	<E -11	<0.001	33/43
	-				Carboxylic acid metabolism	<E -18	<0.001	134/258
	Mitochondrial matrix	<E -7	0.001	64/150	-			
	Redox activity	<E -8	<0.001	93/228	Redox activity	<E -17	<0.001	107/197
	Mitochondrial ribosome	<E -5	0.01	36/78	-			
	-				Ribosome biogenesis	<E -14	<0.001	97/182
	Proteasome complex	<E -8	<0.001	28/43	Proteasome complex	<E -13	<0.001	36/45
	Nucleotide metabolism	<E -5	0.044	35/79	Nucleotide metabolism	<E -11	<0.001	49/79
	Endoplasmic reticulum	<E -7	0.001	127/356	Endoplasmic reticulum	<E -07	<0.001	118/290
	Hexose catabolism	<E -5	0.007	17/26	Hexose catabolism	<E -06	0.005	18/26
	Protein folding	<E -6	0.001	33/62	-			
	-				Cell wall	<E -6	0.001	29/50
**Low correlation**								
Low level in RNA (4/5), high in protein (1/2)	Ribosome biogenesis	<E -5	0.022	24/190				
	Spore wall assembly	<E -6	0.006	10/35				
	Glycoprotein biosynthesis	<E -5	0.01	13/66				
	Oxidoreductase activity, acting on the CH-CH group	<E -5	0.019	5/9				
	Protein amino acid glycosylation	<E -5	0.046	12/62				
Low level in protein (4/5), high in RNA (1/2)	-				Membrane	< E -6	0.001	46/665
	-				Transporter activity	< E -6	0.001	24/246
	-				Cell wall	< E -6	0.002	10/50
	-				Vacuole	< E -5	0.012	15/128

*Comparisons were done as in Figure 2. Then, the genes corresponding to different levels of correlation were divided into groups according to their expression level and the GOs were searched. Only statistically significant categories are shown. High correlation class includes both very high and high correlation classes from Figure 2b, while the low correlations include both low and very low correlations classes, also from Figure 2b. ^†^Absolute *p *value. ^‡^Adjusted *p *value. ^§^The number of genes shows how many of the genes in the GO category present among the genes analyzed in each pairwise comparison are within the selected quintile.

Some GO categories also appeared significantly over-represented in the 'low correlation' classes, thus involving comparisons between variables from quintiles 4/5 and quintiles 1/2: ribosome biogenesis, spore wall assembly, glycoprotein biosynthesis, and so on, for the high TR/low TLRi; and membrane, transporter, and so on, for the high RA/low PA (Table 1). It is interesting to note that 24 genes from the 'ribosome biogenesis' category (Additional data file 9) appeared in this class as well as in the very high correlation class described above. This means that these genes have very high amounts of mRNA and protein, a high TLR but a low TR. These last results indicate that some genes use opposite strategies for mRNA and protein molecules, revealing the existence of several different expression strategies for yeast genes.

### Clustering of yeast genes according to the six variables of gene expression

The previous results suggest that functionally related genes tend to be grouped according to their gene expression variables. To further explore this possibility, we performed a clustering analysis of the 3,991 genes for which data on at least 5 variables were available (Additional data file 13) as a function of their RA, PA, TR, TLRi, RS and PS values. We could have used TLR instead of TLRi, but we chose to use TLRi here because it is not mathematically linked to RA, thus making the clustering less prone to artifacts. In any case, using different normalization methods, or using TLR instead of TLRi, led to essentially similar results (not shown). Since the value ranges for the six variables were quite different, we used the z-score normalization because it better preserves the original relative dispersion. As a result, each gene was characterized by a profile for the arbitrarily ordered (1 to 6: RA-TR-RS-PA-TLRi-PS) variables, which allowed comparing all the genes for common profiles using standard clustering methods. For this we chose the Self-organizing Tree Algorithm (SOTA) [25] from the GEPAS package [26]. This is a self-organizing neural network that expands depending on the relationships among the units being analyzed. The growth nature of this procedure allows it to be stopped at the desired level of similarity resolution, which is reflected in a higher or lower number of clusters.

**Figure 3:**
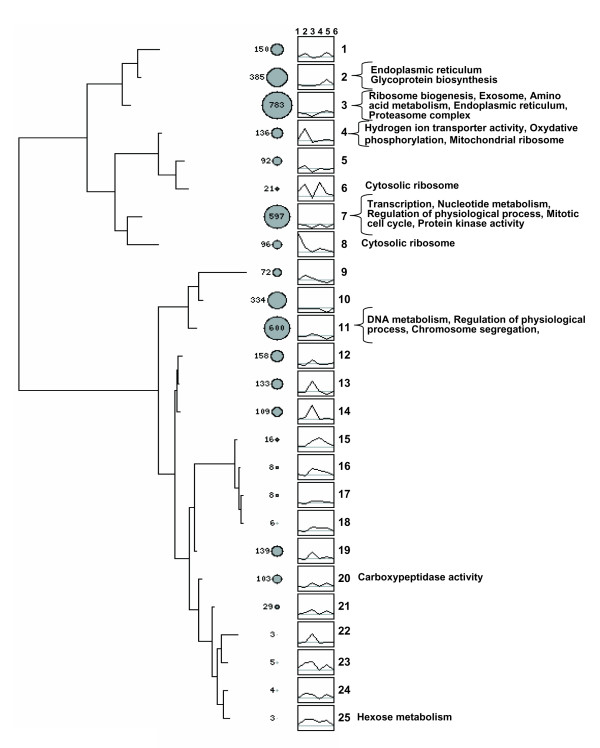
Cluster analysis of the z-score values for the six variables. A SOTA dendrogram is shown. Circle size and the number to the left of the circles indicate gene cluster size. Each gene is characterized by a profile arbitrarily ordered (1 to 6) as RA-TR-RS-PA-TLRi-PS that allows comparison of all the genes for similar profiles. In the right margin of the tree the GO terms that appear significantly over-represented among the genes contained in the corresponding cluster(s) are indicated. The complete list of GO terms and *p *values is given in Additional data file 9. Note that clusters 1-8 correspond to genes showing prevalence of stabilities over synthesis rates and that the second large branching (clusters 9-25) corresponds to genes showing a prevalence of RS (variable 3) over TR (variable 2). The grey line in each cluster graph corresponds to zero. The horizontal branch length reflects the degree of variability between clusters.

Figure 3 shows the dendrogram obtained by using a variability threshold, which produced 25 clusters with this data set. Other variability thresholds generating different numbers of clusters were also considered (Additional data file 3) but the main groupings discussed below were found consistently. The clusters obtained are represented by an average profile that describes the relationships between the six variables for a group of genes. The overall branching pattern of the tree generated was characterized by two large groups: in one of them (clusters 1-8) most clusters showed profiles in which rates (points 2 and 5 in the profile) were higher than stabilities (points 3 and 6). These clusters were enriched mainly in genes coding for subunits of large macromolecular complexes, such as cytosolic and mitochondrial ribosomes and the proteasome. The absolute *p *values were strikingly more significant than in the second group (Additional data file 10); for example, cluster 8 had 72 of the 125 cytosolic ribosome genes analyzed with a *p *value of 10^-98^. Ribosome biogenesis (cluster 3, *p *= 10^-22^), amino acid metabolism (cluster 3, *p *= 10^-7^), transcription (cluster 7, *p *= 10^-11^), and mitochondrial ribosome (cluster 4, *p *= 10^-5^) were other highly significant categories. The second large group included clusters in which RS tended to be higher than TR. These clusters (11-23) were enriched in several GO categories with relatively low *p *values: DNA metabolism (cluster 11, *p *= 10^-5^), chromosome segregation (cluster 11, *p *= 10^-5^), and carboxypeptidase (cluster 20, *p *= 10^-5^) were the most relevant. Additional levels of variability-based clustering were investigated using the CAAT program [26]. This method allows selecting the best clustering level according to variability parameters and then looking for statistically significant GO categories. The analysis resulted in the finding of additional clusters at both higher and lower levels than those shown in Figure 3. For instance, clusters 3, 7 and 11 could be split into smaller ones (Additional data files 4, 5 and 6) to which some specific categories could be assigned.

The finding of many groups of functionally related genes or whose proteins form macromolecular complexes clustering together suggests that the yeast *S. cerevisiae *uses CES in order to coordinate its physiological functions.

### Detailed analysis of functional groups

**Figure 4:**
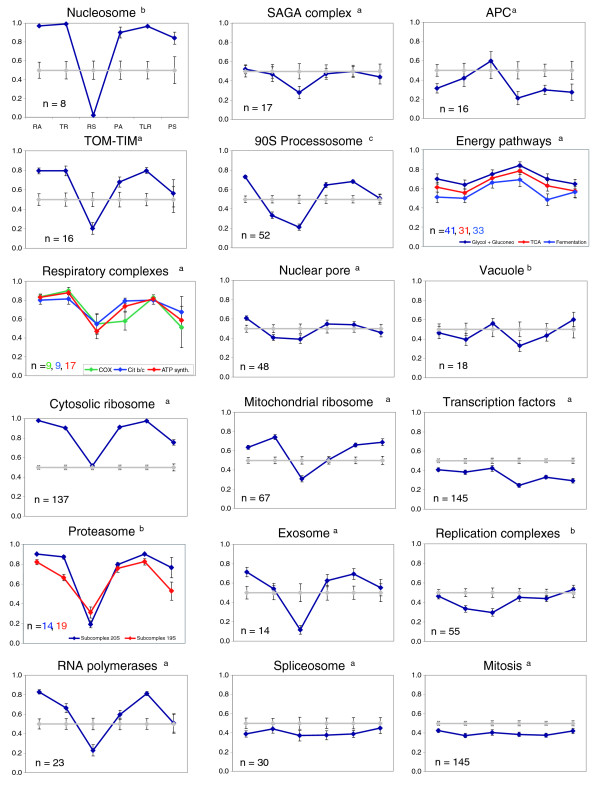
Average 6VP for some functional groups. The color lines represent average rank values for each variable. Grey lines represent average values of 1,000 random samplings with the same sample size as the analyzed functional group. They have been omitted in some graphs for clarity. Bars in the graphs represent the standard error. n, indicates the number of genes in each group. Some additional 6VP graphs are shown in Additional data file 5. Sources for the different groups are: a, GO categories; b, MIPS complexes; c, Straub *et al*. [40].

Since many clusters in Figure 3 contained functionally related genes, we hypothesized that the profiles described above could be taken as signatures of the corresponding CES. Given the appearance of macromolecular complexes as significant categories, we performed a supervised analysis of some of the stable complexes of the Munich Information Center for Protein Sequences (MIPS) list and other GO categories. Figure 4 shows the profiles, in this case using percentile order and TLR, of some biologically relevant groups. We used percentile order to better show features for each functional group. The TLR was selected here instead of TLRi because it reveals better the relative importance of rate and stability in the final PA. The graphs represent the average value of the percentile for each variable and its associated standard error. We denote this signature profile as 6VP. A distinctive common pattern could be clearly observed for some groups. These were those tending to have values for TR and TLR higher than RS and PS (rates higher than stabilities) and corresponded to stable macromolecular complexes. The error associated with each variable was always lower than that expected for a group of the same number of randomly selected genes. This can be seen by comparing the error bars for each variable in each group (color) with the error bars of random groups (grey). A list of numerical average values for each group and the random control can be seen in Additional data file 12. The most relevant feature was that relative RS was always lower than RA and TR. Only some specific complexes (for example, anaphase promoting complex (APC), spliceosome) had a different pattern. Other functionally related groups, not forming stoichiometric complexes, had RS similar or higher than TR (right column in Figure 4; the genes in these groups were included in clusters 11-24 in Figure 3). There seemed to be no obvious relationship between biological noise (DM, as calculated by Newman *et al*. [4]) and the kind of 6VP (results not shown). Cytosolic ribosomal proteins were one of the most uniform groups (Figures 3 and 4). Nevertheless, as shown also in Figure 3, six genes encoding proteins of this group showed a variant profile characterized by an inversion of the respective levels of TR and RA (cluster 6). We have not been able to put forward an explanation for the variant pattern observed in those ribosomal proteins.

### Comparison of mRNA and protein patterns

The plots in Figures 3 and 4 show that mRNA variables (points 1-3) were less balanced than those of the protein. To test whether this is a feature of only some groups or a general characteristic of yeast gene profiles, we made several statistical analyses using TLR data.

First, given that RS seemed to be lower than TR for many groups, we analyzed the whole gene set (Table 2). Although genes with TR > RS were slightly more abundant than expected, the difference was not statistically significant. However, it is true that genes with a lower TR than RS were less common than expected and that those for which TR = RS were more frequent than expected. This trend was more marked when using only genes from the MIPS set of protein complexes. The analyses for protein profiles showed that they tended to be less unbalanced than those of mRNA, with a highly significant excess of genes with TLR = PS. This prompted us to analyze the whole profiles, including amounts of both products (RA and PA). It can be seen in Table 3 that both mRNA and protein had a significant excess of flat profiles, although this effect was much more important for protein. Similar results were obtained classifying genes into ten instead of five categories (results not shown).

**Table 2:** Statistical analyses for predominance of rates or stabilities in protein or mRNAs

	Total		MIPS complexes	
	
Pattern	Observed	Expected	Observed	Expected
TR > RS	1050 (24.6%)	1025 (24%)	**454 (27.1%)**	402 (24%)
TR < RS	*925 (21.7%)*	1025 (24%)	*331 (19.8%)*	402 (24%)
TR = RS	**2296 (53.8%)**	2221 (52%)	**891 (53.2%)**	872 (52%)
TLR > PS	*722 (21.6%)*	802 (24%)	*316 (21.5%)*	352 (24%)
TLR < PS	*539 (16.1%)*	802 (24%)	*212 (14.4%)*	352 (24%)
TLR = PS	**2080 (62.3%)**	1737 (52%)	**941 (64.1%)**	765 (52%)

Statistically significant observed values are highlighted: bold, over-represented; italics, under-represented.

**Table 3:** Analyses of the flatness of the patterns

Pattern	Observed	Expected
Flat RNA	**1182 (27.69%)**	990 (23.2%)
Non-flat RNA	*3086 (72.30%)*	3278 (76.8%)
		
Flat protein	**1371 (42.8%)**	720 (23.2%)
Non-flat protein	*1731 (57.2%)*	2382 (76.8%)

Statistically significant observed values are highlighted: bold, over-represented; italics, under-represented.

**Figure 5:**
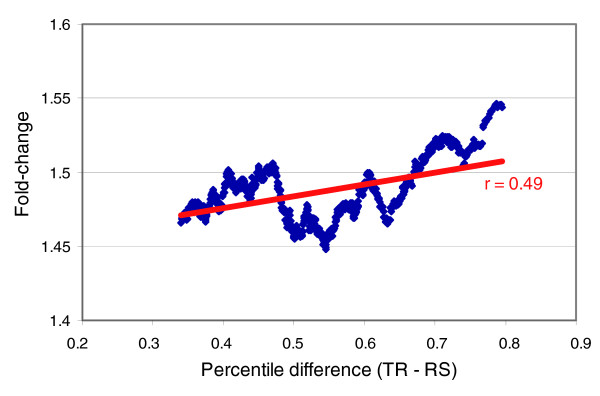
Analysis of transcriptional regulation. Sliding window representation of absolute percentile difference between TR and RS variables (x-axis) against fold-change average (y-axis) from comprehensive expression analysis of stress conditions by Gasch *et al*. [14] for the 1,050 genes having a prevalence of TR over RS after quintile subdivision (Table 2). The width of the window was 200 genes.

The fact that mRNA profiles were more unbalanced than protein ones could be a consequence of strategies favoring regulation at the transcription level. To test this hypothesis, we calculated the average fold-change of yeast genes in the study of Gasch *et al*. [14] in which cells were analyzed under many different conditions that favored changes in gene expression. It can be seen in Figure 5 that the increase in the difference TR - RS tends to be positively correlated with fold-change. The slope of the graph is significantly different from 0 (b = 0.080; standard error = 0.005; t = 16.24; *p *< 0.001).

## Discussion

The yeast *S. cerevisiae *is considered to be the first organism for which a comprehensive description of most gene products and their functional integration will be obtained [27]. The reason for this is that functional genomics methods are providing systematic information about many steps in the pathways of gene expression flow. In this organism, for the first time in biology, there are estimates of the amounts of protein and mRNA as well as their synthesis rates and stabilities at a genomic scale. We have used data previously published by our [19] and other groups [8,9,17,18,20,22] for TR, RA, RS, PA, TLRi and PS together with our computations from previous experimental data [20] of TLR. As a result, we have obtained comprehensive information about the genetic expression flow for 5,968 yeast genes (Additional data files 8 and 13), with at least two of the above variables being compared.

As indicated previously, the quality of the data used in this analysis was variable. For instance, RA data calculated from DNA microarrays are thought not to be reliable below approximately 1 molecule/cell [28]. PA data are probably even less accurate [8]. As discussed by Jansen and Gerstein [29], functional genomics data sets contain a high degree of experimental uncertainty because they have a high amount of error and noise. The use of these data sets can also be hampered because the results were obtained by different laboratories under non-identical growth conditions. We decided to use normalized data to avoid problems related to the uncertainty of absolute values and the comparison of data measured in different scales. Since experimental error and noise should randomize the data, then no statistically significant results should be expected after analyses such as ours. However, our results demonstrate that, even using data from diverse sources, global analyses can benefit from the integration of many data, leading to biologically meaningful conclusions.

To our knowledge, no previous studies have performed exhaustive comparisons among these variables as described here. Single comparisons between RA and PA in yeast have been done previously [4,8,9,11-13,17,18,30]. Correlation coefficients were significant but not very high. For some groups of genes the correlation is low, which has been interpreted as an indication of post-transcriptional regulation [11]. Nevertheless, there are important differences between different functional groups. The general conclusion of these simple comparisons was that there is a significant positive correlation between the amount of a protein and that of the mRNA encoding it. We postulate here that it is mainly due to the coordination between their synthesis rates (see below). We previously made a simple comparison between TR and RA [19]. The positive correlation found was not unexpected because it is commonly accepted that mRNA amounts depend directly on their synthesis rates. Beyer *et al*. [17] performed a different kind of analysis, centered on functional categories, of the TLR-PA comparison. TLR can change depending on the RA but also independently of it in some genes [10]. Belle *et al*. [9] also made a comparison between PS, TLR and PA. They found positive correlations between PA and the other two variables. Lu *et al*., [11] made comparisons between PA and TR, TLR and TLRi. They found positive correlations in all cases.

We have explored several ways to normalize the data before comparing them. For correlation analysis we chose to rank every variable because, in this way, the relative position within the cell physiology of each gene allows an easier analysis of the positions of specific GO classes. We have found that, apart from confirming the positive correlations cited above, there is a significant, high positive correlation between TLRi and TR. Since RS and PS are not correlated (Figure 2a), it can be concluded that the main determinant of the observed correlation between the amounts of mRNA and protein is the coordination of their synthesis rates.

The negative correlation between RA and RS is interesting. Wang *et al*. [22] did not find any correlation using similar data. This could be due to their use of Pearson correlation whereas we have used Spearman rank correlation, which is less sensitive to noise in individual data sets. A negative correlation like this one has been observed for *Escherichia coli *[30] and for the archaeon *Sulfolobus *[31]. The low mRNA stability of highly transcribed genes in these organisms was partially interpreted as a feature for noise minimization and a way for rapid adaptation to environmental changes. Here, we have found a negative correlation between RS and TR in *S. cerevisiae*. Thus, it seems likely that free-living organisms use similar strategies with regard to mRNA stability.

A negative correlation between TLR and RS was also found. Because TLR is the product of TLRi and RA, this can be the result of the negative correlation of RA and RS and the lack of correlation between TLRi and RS. However, no correlation between RS and TR and a positive correlation between ribosome density and ribosome occupancy (both components of TLRi) and RS [15] have been found in *S. pombe*. We do not know whether this reflects a truly different behavior between these two yeast species or it is due to the small and biased number of mRNAs (only the 868 least stable ones) for which RS was calculated in that study.

To further verify the consistency of the groupings obtained with these analyses, we tried different clustering methods. For clustering analysis we assayed several normalization procedures, including ranking and a range of normalizing transformations, and different clustering methods: PCA, k-means, and hierarchical unsupervised growing neural networks. We found that z-score normalization and SOTA hierarchical clustering [25,26] produced the best results in terms of recovery of significant GO categories. This reasoning is considered to be the best method to evaluate the quality of clustering protocols [32]. In any case, the general conclusions obtained after clustering were the same regardless of the algorithm used. We are aware that our method has an unavoidable bias due to the identical weight assigned to the six variables, but this affects similarly all categories found and, in consequence, cannot produce biases in the recovery of GO categories.

The SOTA clustering of z-score vectors for the 3,991 genes considered (Additional data file 13) yielded a tree with two main subgroups (Figure 3). Many clusters were enriched in specific GO categories (Additional data file 9). The relative relevance of each variable is reflected in the different profiles, in which, because of the z-score normalization, the six variables are directly comparable. Moreover, since the analysis has been made for all the genes simultaneously, it demonstrates that functionally related genes tend to use similar strategies for their expression. We have introduced the acronym CES to denote this observation.

Clusters in the upper part of the tree in Figure 3 contain many more significant GO categories and with higher significant *p *values than those in the lower part. The 6VP defining the upper clusters are characterized by synthesis rates for either mRNA, protein or both ranking higher than the corresponding stabilities. These clusters are enriched in GO terms for large, stoichiometric and stable cellular protein complexes, such as cytosolic ribosome, mitochondrial ribosome or proteasome. Some of these groups are specifically analyzed in Figure 4. Histones represent one of the most extreme behaviors. Their profile (nucleosome) is similar to others in the 'higher rates' branch from Figure 3. They show extremely high synthesis rates and amounts of mRNA and protein and low RS. However, the small size of this group (eight genes) precludes statistical relevance. Other protein complexes, such as TOM-TIM, ATP synthase, RNA polymerases, cytosolic ribosome, exosome and proteasome, have a 6VP of the same type, but not so strongly marked. The mitochondrial ribosome, the 90S processosome (Figure 4) and the vacuolar ATPase (Additional data file 7) also show slight variations from that common 6VP profile. Some other GO categories not forming stable complexes, such as 'translation' and 'glycoprotein biosynthesis', also have a 6VP similar to this one (Additional data file 7).

Using the MIPS classification, we found an enrichment of genes belonging to protein complexes in the profiles with a predominance of TR over RS (Table 2). It is accepted that proteins belonging to the same complex must be present in similar amounts because the excess of any subunit would be wasteful (see [33]). Therefore, coordination of the corresponding PAs is to be expected. However, many (perhaps all) protein complexes in the cell are formed by subunits that are not exclusive to only one complex, being included in other complex(es) as well. Some studies on yeast complexes have shown that a core or protein sub-complex of highly co-expressed and functionally related subunits exists and that this core is surrounded by less cross-related, 'halo' proteins [33,34]. Additionally, some complexes are transient while others are permanent [33]. Our results show that the large and permanent complexes correspond to the best-clustered groups and that they tend to have higher TR than RS. Fraser *et al*. [3] found that genes belonging to protein complexes have less biological noise than average because of a high TR and low number of 'transcriptions per mRNA', which implies low RS. Thus, it seems that one reason for common 6VPs in members of some complexes could be the need for low noise. Previously, it has been found in some studies that genes for the cytosolic and mitochondrial ribosomes and the proteasome subunits behave coordinately with respect to TR, RA and/or RS [19,23,33]. On the other hand, Wang *et al*. [22] found that subunits of the main cellular complexes, including both kinds of ribosomes, the nucleosome and the proteasome, have similar RS. We have found that other variables, such as PA and TLR, are also conserved for such complexes. We can conclude that, in general, the whole 6VP is very uniform for the members of these permanent complexes. This result is also observed for other smaller complexes (Figure 4) and for other functionally related genes also found in the clusters obtained with the SOTA algorithm (Figure 3).

The predominance of rates over stabilities (especially TR over RS) shown by the groups in the upper part of the tree (Figures 3 and 4) is a strategy that favors speed over economy in the response, because the amount of the macromolecule is controlled by relatively high rates of synthesis and degradation. This strategy has a higher energy cost but it allows rapid responses due to the relatively low stability of the macromolecule. It is, perhaps, more useful for free-living organisms than for higher eukaryotes, since the latter have evolved many other physiological mechanisms to rapidly adapt to changing environmental conditions. A prediction of this hypothesis is that genes with TR > RS will be significantly more regulated than the average. This is the actual result we found using data from a set of 142 experiments [14] in which a large set of changing growth conditions was analyzed (Figure 5). The trend for genes to be more regulated at the transcriptional level seems to be more pronounced for those having a TR - RS difference higher than 0.5. In any case, strategies with a prevalence of rates for protein (TLR > PS) and mRNA (TR > RS) are not frequent among the whole set of yeast genes. In fact, the number of genes with TLR > PS is lower than expected (Table 2).

The lower part of the tree in Figure 3 is enriched in some GO categories. The common signature of these groups is that their RS is higher than the corresponding TR. This strategy is less common than expected by chance, especially for genes belonging to MIPS complexes (Table 2) and it represents an opposite strategy to that used by the groups described above. It favors economy over speed in the response at the mRNA level. This would be appropriate for genes that should not have to respond rapidly or that are regulated post-transcriptionally or, even, post-translationally, such as most metabolic enzymes.

It is interesting to analyze in more detail the group 'Energy pathways' in Figure 4. It comprises a set of very abundant proteins from the functional categories 'TCA cycle', 'glycolysis and gluconeogenesis' and 'fermentation' that behave similarly. Their PA is almost at the level of cytosolic ribosome proteins. However, they present a totally different strategy. Abundant mRNAs are obtained using a lower TR than for ribosomal proteins but with quite high messenger stabilities. In fact, the GO categories related to energy derivation from carbohydrates are the ones showing the highest RS (Additional data file 13). It is clear that the cell spends much less energy maintaining the level of these mRNAs. The price would be that their RAs change more slowly, but this might not be a priority for the cell. Energy generation processes are almost equally necessary at all times. A prediction is that this kind of 6VP will be more common in higher eukaryotes for the same reasons pointed to above. Some other groups have very low PS compared to TLR, such as TOM-TIM, RNA polymerases, cytochrome oxidase, 19S proteasome (Figure 4), as well as the glycoprotein biosynthesis, translation and vacuolar ATPase (Additional data file 7). Therefore, using the same reasoning as for transcription, their corresponding genes are also candidates to be regulated at the translational level.

To obtain the desired RA or PA, the most important factor seems to be the synthesis rate. This is reflected in the positive correlations observed between RA, PA, TR and TLR (Figure 2). However, mRNA is not the final goal of the gene expression, a role that corresponds to the protein. This establishes a clearly different role for mRNA and protein in gene expression. Our comparisons show that, in yeast, these different roles can be mirrored by the different behavior of protein and mRNA sub-profiles and, especially, by the different behaviors of RS and PS.

It seems that whereas PS works in the same direction as TLR to control PA, which is, therefore, positively correlated with amounts and rates, RS works in the opposite direction for most genes. Among the possible expression strategies, those with less stable molecules are more costly but allow faster tracking of environmental changes [24]. In this way, strategies with relative low RS or PS are only appropriate for genes expected to need rapid expression changes. The costs for low RS and for low PS are, however, very different. Translation requires much more energy than transcription. For a standard yeast gene, transcription consumes six ATP molecules per triplet for a mRNA molecule, while translation consumes four ATP molecules per amino acid. However, on average, mRNAs are six times less stable than proteins (26 minutes versus 154 minutes) and the mean number of protein molecules per mRNA molecule for a yeast gene ranges from 4,000 [17] (our data) to 5,600 [11]. This means that costly strategies for mRNA may be economical and efficient if they allow for a fast change in the amounts of mRNA to minimize translation costs. In Figure 4 it can be seen that most cellular complexes formed by abundant proteins (ribosomes, proteasome, nucleosome, and so on) follow strategies characterized by a relatively low RS. All these complexes require a tight regulation of PA because they form abundant cellular machines that are expensive to maintain.

Protein variables show less unbalanced profiles than their mRNA counterparts. The average standard deviation (SD) for PA-TLR-PS, expressed as percentile values, is 0.196 while for mRNA it is 0.235 (Additional data file 13). The smoothness of the protein profile is even more pronounced in the group of very highly correlated genes (Figure 2b), with an average SD of 0.163. Although 'flat' profiles for mRNA are more abundant (27.69%) than expected (23.2%), this is especially striking for proteins (42.8% versus 23.2%; Table 3). All these data indicate that protein variables tend to be less unbalanced than those of mRNA. Whereas this cannot rule out the existence of regulatory mechanisms at the protein level, it clearly indicates that a 'compensatory rate-stability mechanism', common for mRNAs, is not that common for proteins. Moreover, the comparison of mRNA and protein profiles for some groups suggests that there is more regulation of these genes at the transcription (including both TR and RS) level. This has been shown to be the case for ribosomal proteins in yeast, contrary to that found in bacteria, *S. pombe *and mammalian cells (discussed in [6,35,36]). Interestingly, in the evolutionarily distant yeast *S. pombe*, ribosomal protein mRNAs do not belong to the short-lived class [36], opposite to *S. cerevisiae*, which supports the idea that the expression of *S. cerevisiae *genes is mainly controlled at the transcription level [6] whereas in *S. pombe *this is at the translation level [15,36]. Given the similarity to 6VP profiles from the other large protein complexes, we suggest that this could also be the case for many of their components. For instance, it has been described that transcriptional regulation (both TR and RS) controls the genes of the proteasome and 90S processosome [37]. The important role for RS in this kind of regulatory mechanism might explain the surprising finding that RS seems to be tightly coordinated for these protein complexes [22].

## Conclusion

We propose that the analysis of all the variables that affect the flow of gene expression is a useful strategy to investigate the regulatory strategies used by a cell. We conclude from our study that the synthesis rates for both mRNA and protein are the main determinants of the amount of the respective molecules and that yeast cells use CESs for genes acting in the same physiological pathways. This feature is more clearly shown for genes coding for large and stable protein complexes, such as the ribosome or the proteasome. Hence, each functional group can be defined by a 6VP that illustrates the common strategy followed by its members. For many groups whose genes encode subunits of protein complexes, there is a tendency to have relatively unstable mRNAs and a more unbalanced profile for mRNA than for protein, which suggests a stronger regulation at the mRNA level.

Current knowledge from other model organisms, such as *S. pombe *[15], indicates that the CES can be different for specific gene groups in different organisms. We anticipate that differences in CES will be even stronger for the different cell types of higher eukaryotes, a result of the large differences in their living environments.

## Materials and methods

### Selection and features of the original data

Many studies have produced RA data from S288c-type yeast strains growing in YPD medium. For our analyses we chose the reference set constructed by Beyer *et al*. [17], who used 36 microarray experiments normalized and corrected for saturation effects using SAGE data [16]. This data set comprises 6,297 protein-coding genes, with 6,117 genes remaining after filtering dubious open reading frames (classified by the Saccharomyces Genome Database; Additional data file 8). In the case of RA data, as in others described later, we also made several tests using other less refined data sets [19,22]. No major variations in the results obtained were found (not shown). For TR/TRi, the only experimental data set available was obtained using the Genomic Run-On methodology [19]. This data set comprised 5,828 genes (5,669 after filtering). For mRNA stability, several genomic calculations using either drug inhibition of RNA polymerase II or the *rpb1-1 *thermo-sensitive mutant and temperature shift were available. We used the overall RNA data set of [22] but other data sets [19,23] were tested and, again, no relevant differences were found. This data set comprised 4,677 genes (4,544 after filtering). For PA, we used the reference set constructed by Beyer *et al*. [17] using data from several sources. This set included 4,243 genes (4,239 after filtering). For TLRi calculation, we used ribosome density data [17] assuming a constant ribosome speed. To derive TLR values, we multiplied TLRi by the RA data described above. This data set comprised 6,154 genes (5,968 after filtering). Finally, for PS we used the recent set of 3,370 proteins (3,367 after filtering) [9]. The whole data set comprised 6,173 genes, for 3,991 of which there were data on at least 5 of the 6 variables considered (Additional data files 8 and 13).

The quality of the different data sets was variable. RA data are quite robust because they were obtained by averaging results from many different sources and, moreover, they were normalized and corrected [17]. TR, RS and PS data were obtained from a single measurement; however, they were verified by comparison with previously determined individual data for some genes [9,19,22]. TLRi, and consequently TLR, data were obtained by averaging two data sets [17]. TLR data have the problem that they were calculated indirectly by multiplying experimentally determined data (the RA and TLRi data sets). This adds the mathematical error associated with these operations and the disadvantage that TLR and RA are not independent. PA data are the average of data obtained using very different techniques (epitope tagging, multidimensional protein identification technology, and two-dimensional electrophoresis [8,17,18]). In spite of this, PA data are less robust than RA data because they are based on fewer measurements and because the techniques used are less accurate than SAGE and DNA microarrays.

For the analyses, we have used a z-score or a percentile normalization to avoid the high dispersion in the unit ranges among the different variables. In this way data retained their relative magnitude within each variable and were directly comparable across variables, thus reducing computation artifacts and enabling easier comparisons and interpretations.

### Cluster analyses

We have used a range of statistical methods for identifying sets of genes with similar expression patterns. The two main approaches correspond to grouping or classifying genes according to their expression patterns and to represent them in a reduced dimension space. Characteristic global profiles were established by means of cluster analysis using the data set of z-score normalized values for the six variables (as mentioned in the Results section) for a total of 3,991 yeast genes for which data for at least 5 variables were available (Additional data file 8).

For cluster analysis we used the SOTArray tool (included in the Gene Expression Pattern Analysis Suite v 3.0 (GEPAS) [26] from the worldwide web server of the CIPF Bioinformatics Unit) using the linear correlation coefficient among the six-variables vectors as distance between genes. The tree was allowed to grow until producing 20, 25 or 30 clusters. Alternative clustering methods were also applied to the same data set. We used k-means clustering [38] with a variable number of clusters from 2 to 25.

In order to validate the quality of the previous clustering procedure, we used the Cluster Accuracy Analysis Tool (CAAT 1.0), also included in the GEPAS package. We calculated a 'silhouette width' for each internal node. This index represents how well each cluster is separated from its direct sister groups; that is, how close are items contained in this cluster (intracluster distance), and how far they are from the sister clusters (intercluster distance). Values for silhouettes range from -1.0 (very bad split) up to 1.0 (excellent split). Values near 0.0 indicate indifferent split. Cluster subdivision was stopped when the silhouette value was not improved in two consecutive divisions.

### Gene Ontology category searches

To test the potential enrichment in GO categories in the different groupings obtained in this study (clusters from SOTA/CAAT trees, correlation groups, and so on), we used the FuncAssociate server [39], which uses a Monte Carlo simulation approach and accepts only significant GO categories according to their adjusted *p *value (computed from the fraction of 1,000 simulations under the null-hypothesis with the same or smaller *p *value and after correction for multiple simultaneous tests). Only GO categories with an adjusted *p *value below 0.05 were considered to be significant.

### Correlation analyses

In order to test for genes having similar values for a given pair of variables, we ranked and ordered the values for each variable, and divided the distributions in quintiles (note that for each pair-wise comparison, the maximum number of gene pairs was considered; thus, the number of genes in each partition depended on the number of genes present in each comparison). Genes belonging to the upper quintile were numbered as 1, genes from the second quintile were numbered as 2, and so on, down to the lowest variable values, included in the quintile numbered as 5. When comparing two variables we classified genes into five correlation categories depending on their quintile difference. Thus, we established five correlation quality categories: 'very high', for genes having the same quintile value in both variables (five possible combinations); 'high', for genes differing in one quintile unit (eight possible combinations); 'medium', when the quintile difference was 2 (six combinations); 'low', for three unit differences (four combinations); and 'very low' for the cases of quintile differences of four units (two combinations). Searches for enrichment in specific GO categories were performed as described above.

To test the global correlation between all pair-wise combinations of the six variables, Spearman rank correlation coefficients were calculated.

### Six variable profiles

We also investigated whether different functionally related gene groups (MIPS complexes, GO categories, and the processosome complex as defined by Staub *et al*. [40] tended to have similar values in the six variables considered in this study. Thus, we used the rank (percentile) ordered values for the six variables for different related genes. We calculated the average rank value (percentile) and represented these values for the six variables ordered as RA, TR, RS, PA, TLR, PS, yielding a 'profile' for each group studied. We calculated also the standard error associated with each average and represented in the profile as error bars. These values were obtained by random sampling (1,000 replicates) among the genes having data for the six variables. Resampling group sizes were equal to that of genes in each considered group and subsequent computation of average and standard deviations for each variable. An estimation of the average standard deviations (aSE) for the six variables was calculated for each group (Additional data file 12).

### Comparison of mRNA and protein profiles

For comparing mRNA and protein profiles, we used the quintile classification of genes as for correlation analyses (Figure 2b and Additional data file 13). We considered prevalence of a variable over another if they differed in two or more quintiles. Differences of 1 or 0 quintiles were considered to be equal. This was done for all genes and for the genes forming protein complexes according to MIPS. Only complexes with more than two proteins were considered (Additional data file 13). Statistically significant differences between observed and expected values (considering all possible combinations by chance) were established by applying a Chi-square test (Table 2).

Prevalence of flat patterns in mRNA and protein was studied separately by considering a flat pattern when the difference in quintile value among the most extreme variables for each molecule was less than three. Similarly, expected values were established by considering all the possible quintile combinations between the three variables for each molecule, and the statistical significance of the differences was assessed by means of a Chi-square test (Table 3).

### Test for transcriptional regulation

In order to test for the transcriptional regulation level among the genes with a prevalence of TR over RS, we selected the genes for which that premise occurred (1,050 genes with TR > RS from Table 2). We represented in a 200-gene-wide sliding window the average fold-change in many stress conditions in the comprehensive study by Gasch *et al*. [14] versus the percentile difference between TR and RS (TR - RS). The statistical significance of the slope was assessed by means of a *t*-test.

## Abbreviations

6VP, six variable profile; CAAT, Cluster Accuracy Analysis Tool; CES, common expression strategy; GO, gene ontology; MIPS, Munich Information Center for Protein Sequences; PA, protein amount; PS, protein stability; RA, mRNA amount; RS, mRNA stability; SOTA, Self-organizing Tree Algorithm; TLR, translation rate; TLRi, individual translation rate; TR, transcription rate; TRi, individual transcription rate; YPD, yeast extract-peptone-dextrose culture medium.

## Authors' contributions

JP-O conceived the original idea and designed the experiments. JG-M collected and curated the data sets and performed most of the analyses. FG-C performed some of the statistical analyses and supervised the computer methods. JP-O wrote most of the paper, and JG-M wrote the experimental section and FG-C corrected it. All three authors extensively discussed the results and their interpretation and approved the final version.

## Additional data files

The following additional data are available with the online version of this paper. Additional data file 1 is a figure showing a plot of abundance and stability for mRNA and protein molecules. Additional data file 2 is a figure showing clustering similar to that shown in Figure 3 but with 20 clusters. Additional data file 3 is a figure showing clustering similar to that shown in Figure 3 but with 30 clusters. Additional data files 4, 5, 6 are figures showing further analysis of some large clusters (3, 7 and 11, respectively) from Figure 3. Additional data file 7 is a figure showing 6VP for some other functional categories not shown in Figure 4. Additional data file 8 is a table showing a summary of numerical data used in the paper. Additional data file 9 is a table listing ribosome biogenesis genes that appear within the low correlation class in Figure 2b. Additional data file 10 is a table providing the complete list of significant GO categories found in clusters from Figure 3. Additional data file 11 is a table providing the complete list of significant GO categories found in clusters from Additional data files 2 and 3 and not present in Figure 3. Additional data file 12 is a table listing the standard error averages calculated for experimental (aSEe) and random sampling (aSEr) estimations for the functionally related groups from Figure 4. Additional data file 13 is a table listing values for the six variables of the 6,173 genes analyzed. Additional data file 14 the description and comments of the figure shown in additional data file 1.

## Supplementary Material

Additional data file 1 Plot of abundance and stability for mRNA and protein molecules. Click here for file

Additional data file 2 Clustering similar to that shown in Figure 3 but with 20 clusters. Click here for file

Additional data file 3 Clustering similar to that shown in Figure 3 but with 30 clusters. Click here for file

Additional data file 4 Further analysis of cluster 3 from Figure 3. Click here for file

Additional data file 5 Further analysis of cluster 7 from Figure 3. Click here for file

Additional data file 6 Further analysis of cluster 11 from Figure 3. Click here for file

Additional data file 7 6VP for some other functional categories not shown in Figure 4. Click here for file

Additional data file 8 Summary of numerical data used in the paper. Click here for file

Additional data file 9 Ribosome biogenesis genes that appear within the low correlation class in Figure 2b. Click here for file

Additional data file 10 Complete list of significant GO categories found in clusters from Figure 3. Click here for file

Additional data file 11 Complete list of significant GO categories found in clusters from Additional data files 2 and 3 and not present in Figure 3 Click here for file

Additional data file 12 Standard error averages calculated for experimental (aSEe) and random sampling (aSEr) estimations for the functionally related groups from Figure 4 Click here for file

Additional data file 13 Values for the six variables of the 6,173 genes analyzed. Click here for file

Additional data file 14 Description and comments of the data shown in additional data file 1. Click here for file
