# Supplementary figures and images for: Common gene expression strategies revealed by genome-wide analysis in yeast
Source: Genome Biol. 2007 Oct 19;8(10):R222. doi: 10.1186/gb-2007-8-10-r222 (PMC2246296; doi:10.1186/gb-2007-8-10-r222)

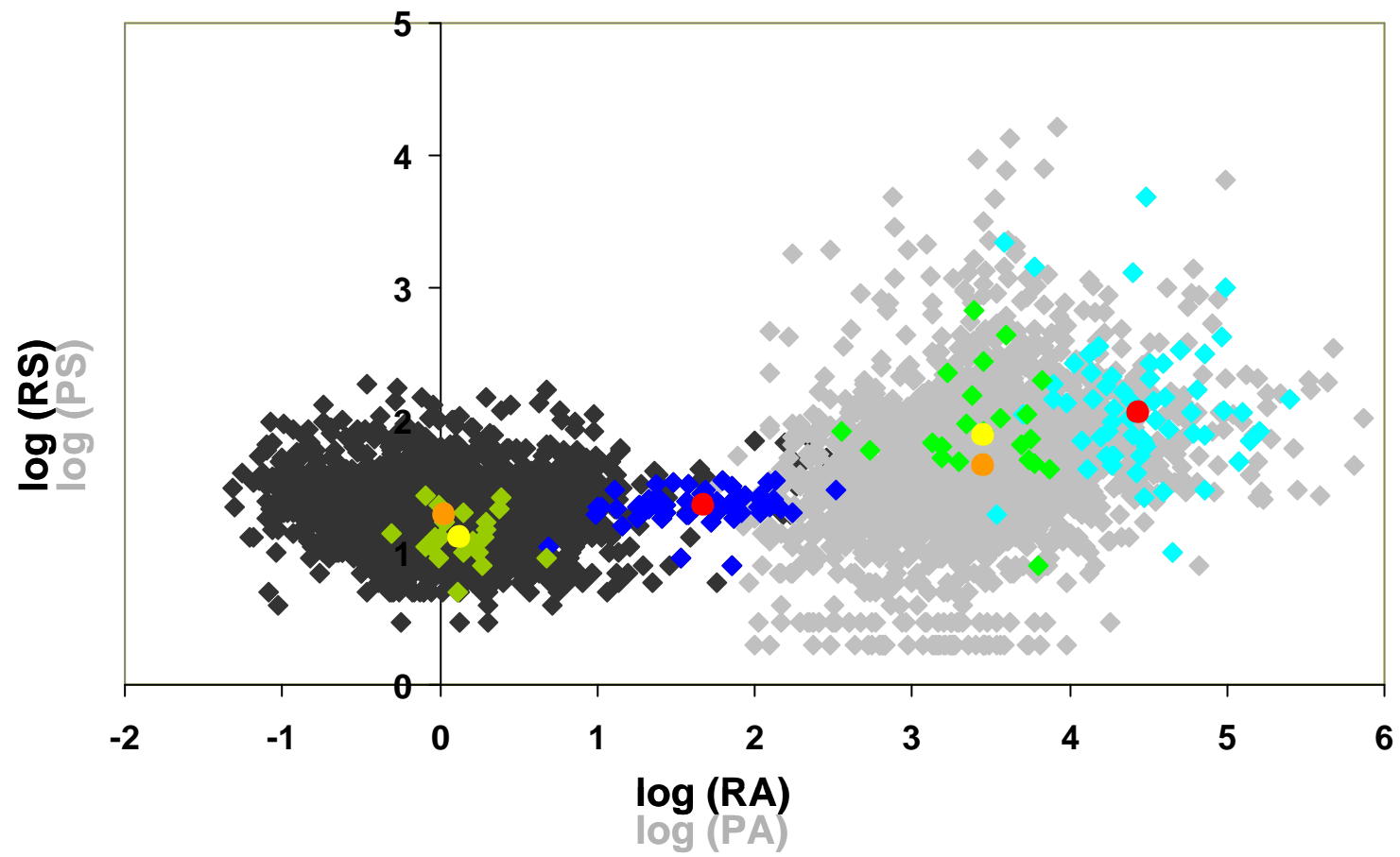

Figure S1

Supplement: Additional data file 1 — Plot of abundance and stability for mRNA and protein molecules. [file gb-2007-8-10-r222-S1.pdf]

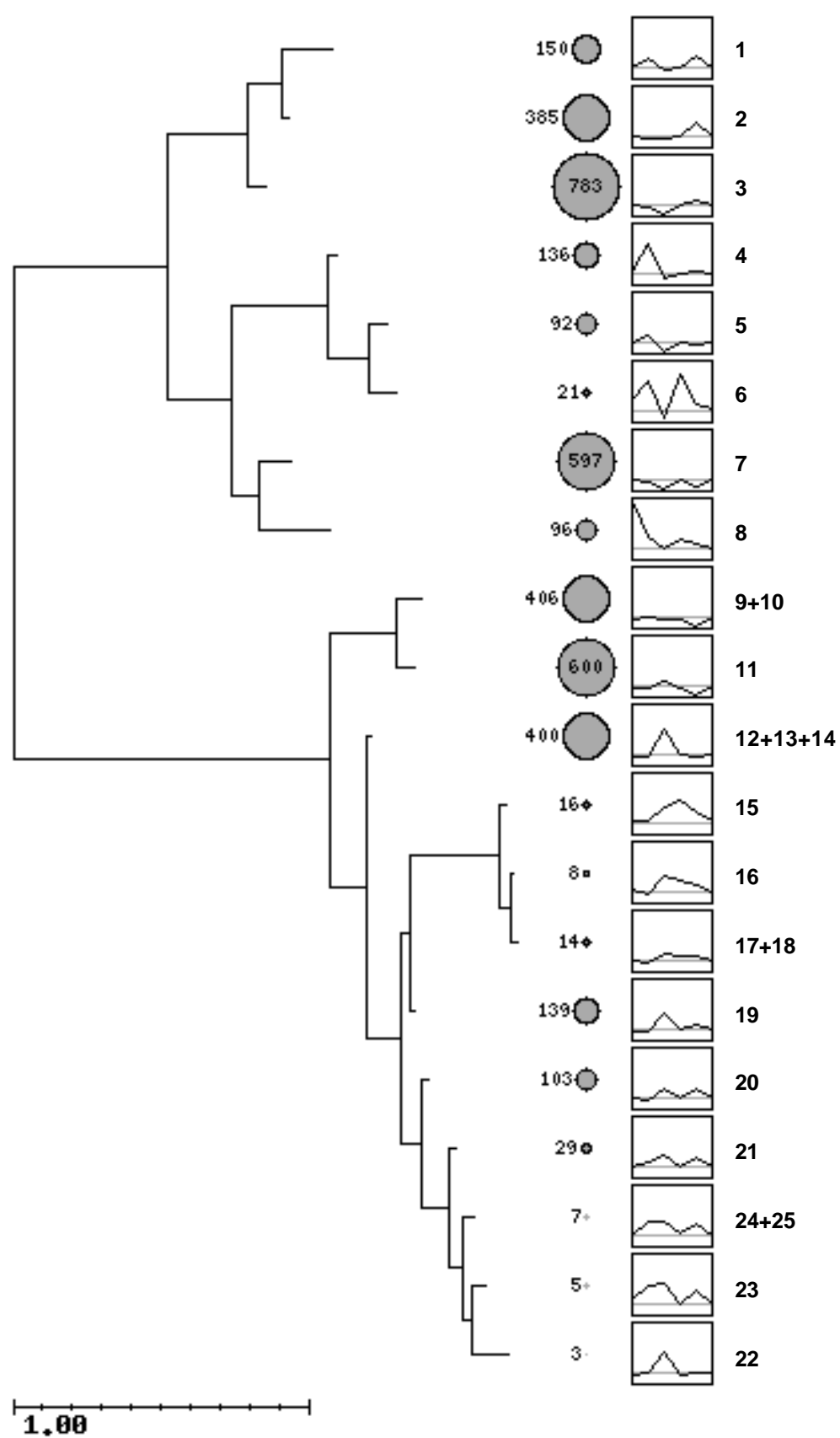

**Figure S2**

Supplement: Additional data file 2 — Clustering similar to that shown in Figure 3 but with 20 clusters. [file gb-2007-8-10-r222-S2.pdf]

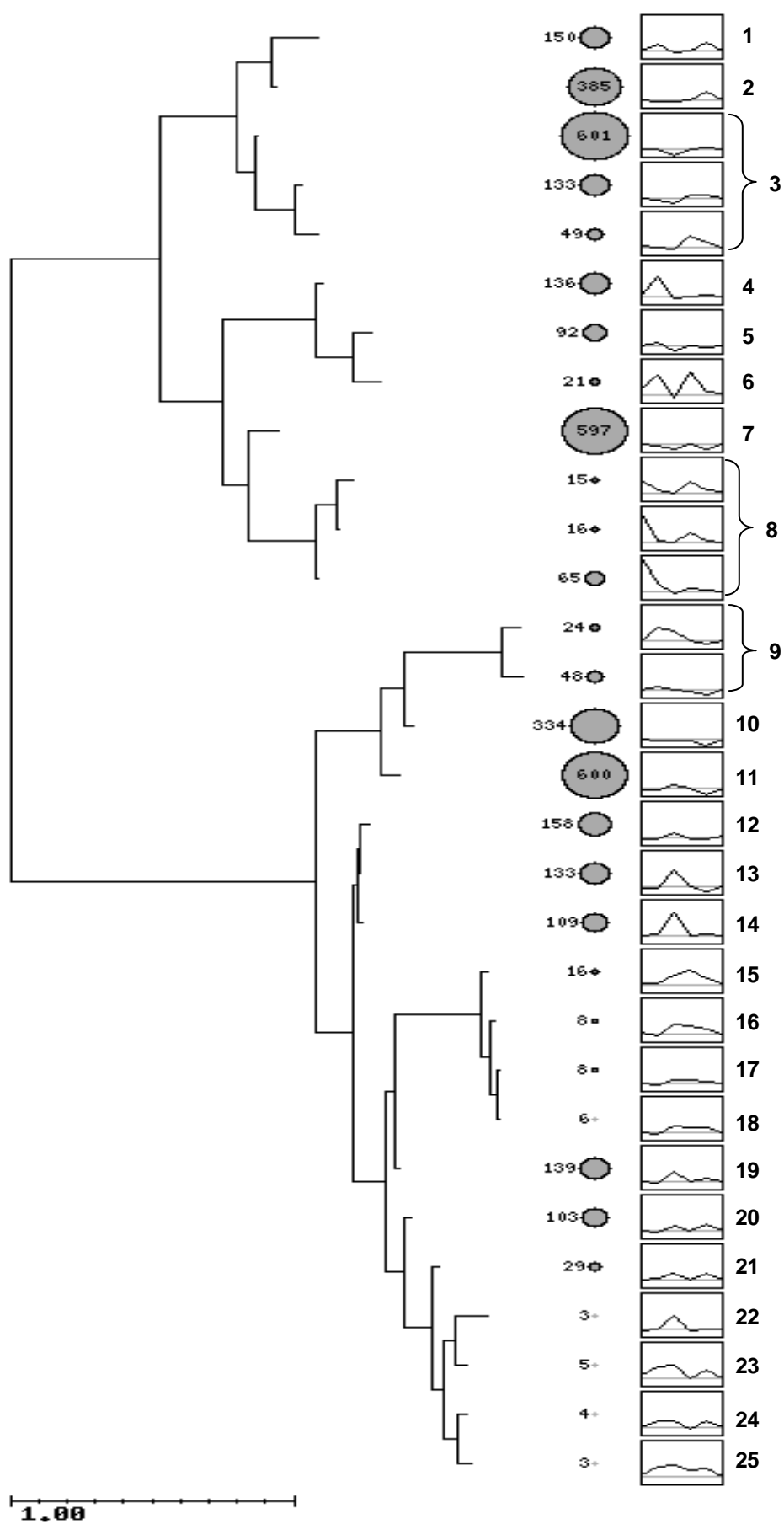

**Figure S3**

Supplement: Additional data file 3 — Clustering similar to that shown in Figure 3 but with 30 clusters. [file gb-2007-8-10-r222-S3.pdf]

## Cluster 3

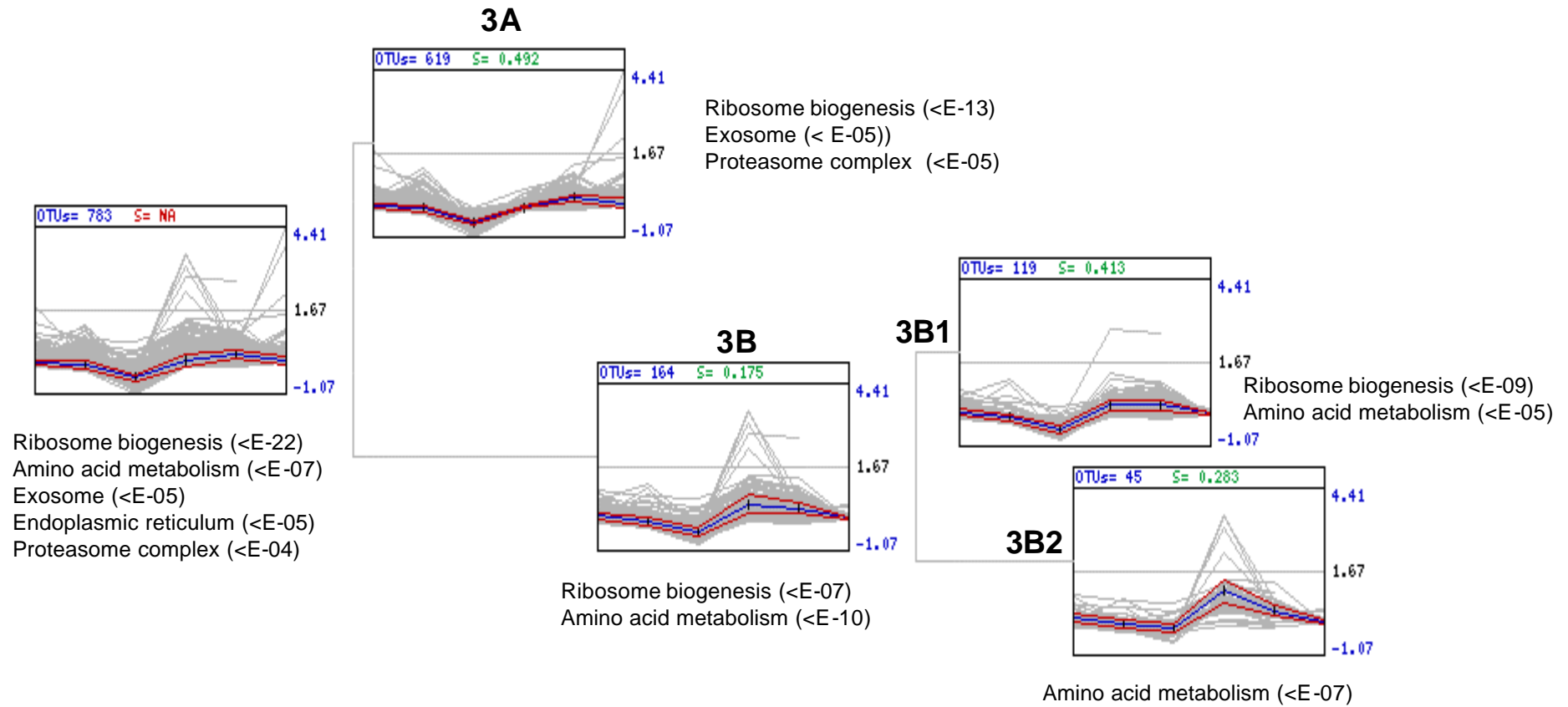

**Figure S4-A**

Supplement: Additional data file 4 — Further analysis of cluster 3 from Figure 3. [file gb-2007-8-10-r222-S4.pdf]

## Cluster 7

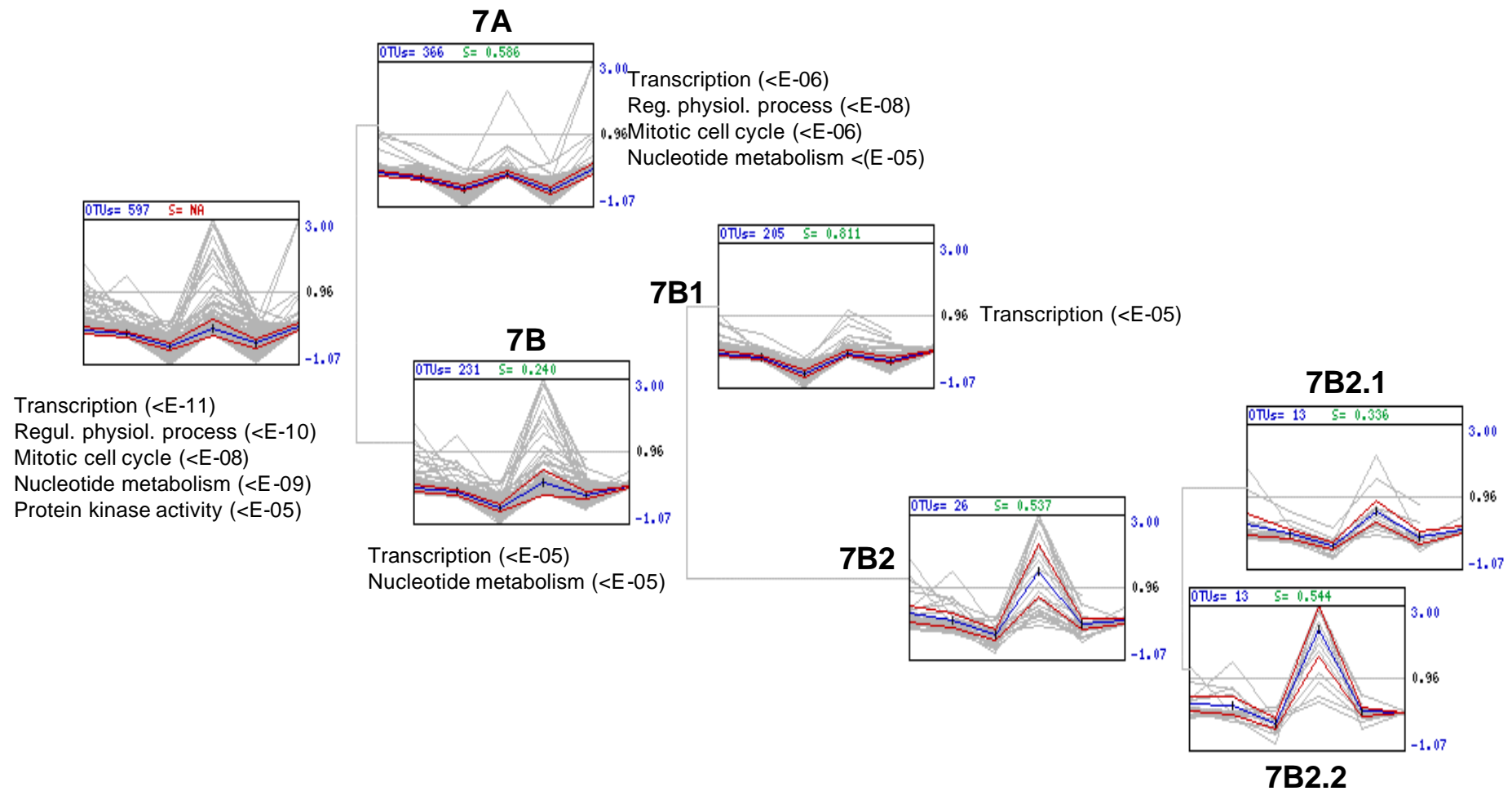

Figure S4-B

Supplement: Additional data file 5 — Further analysis of cluster 7 from Figure 3. [file gb-2007-8-10-r222-S5.pdf]

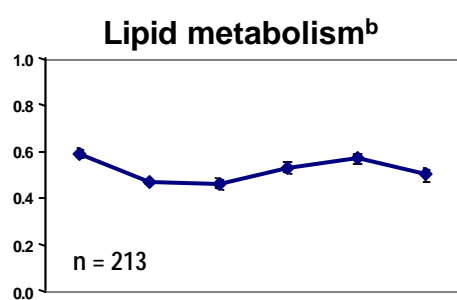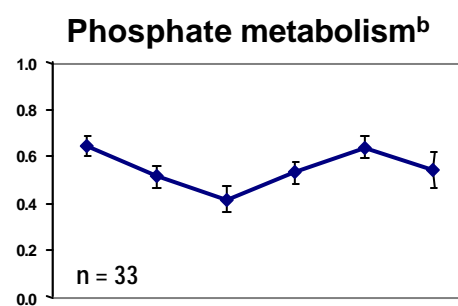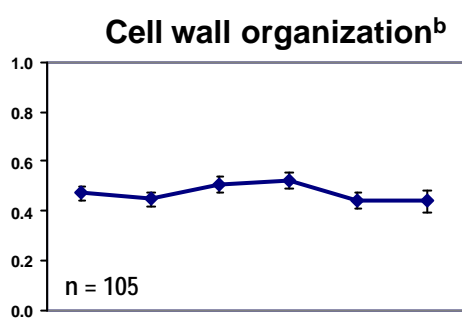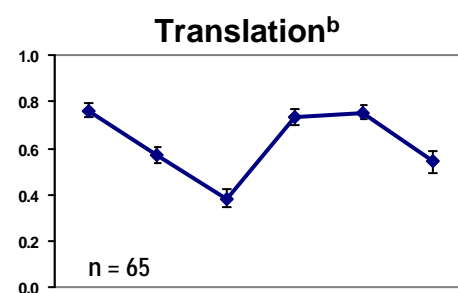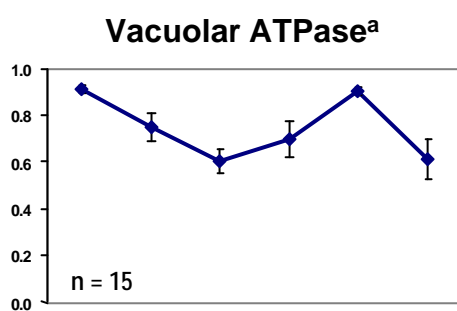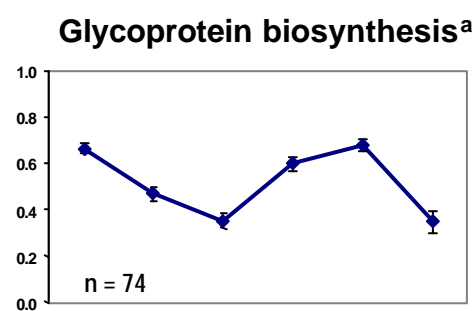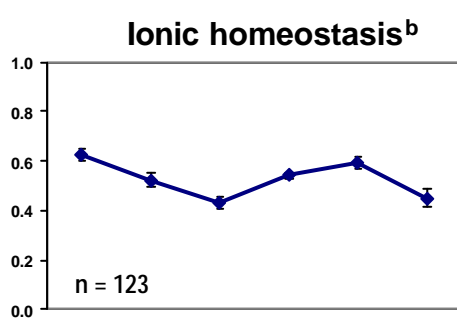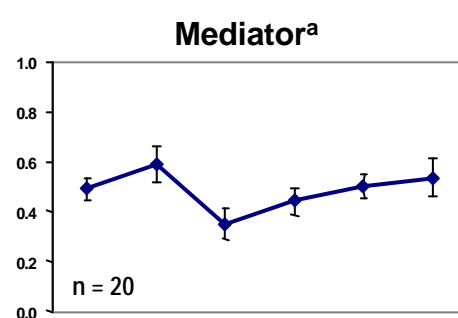

**Figure S5**

Supplement: Additional data file 7 — 6VP for some other functional categories not shown in Figure 4. [file gb-2007-8-10-r222-S7.pdf]
