# Supplementary material for: Common gene expression strategies revealed by genome-wide analysis in yeast
Source: Genome Biol. 2007 Oct 19;8(10):R222. doi: 10.1186/gb-2007-8-10-r222 (PMC2246296; doi:10.1186/gb-2007-8-10-r222)
Supplement: Additional data file 6 — Further analysis of cluster 11 from Figure 3. [file gb-2007-8-10-r222-S6.pdf]

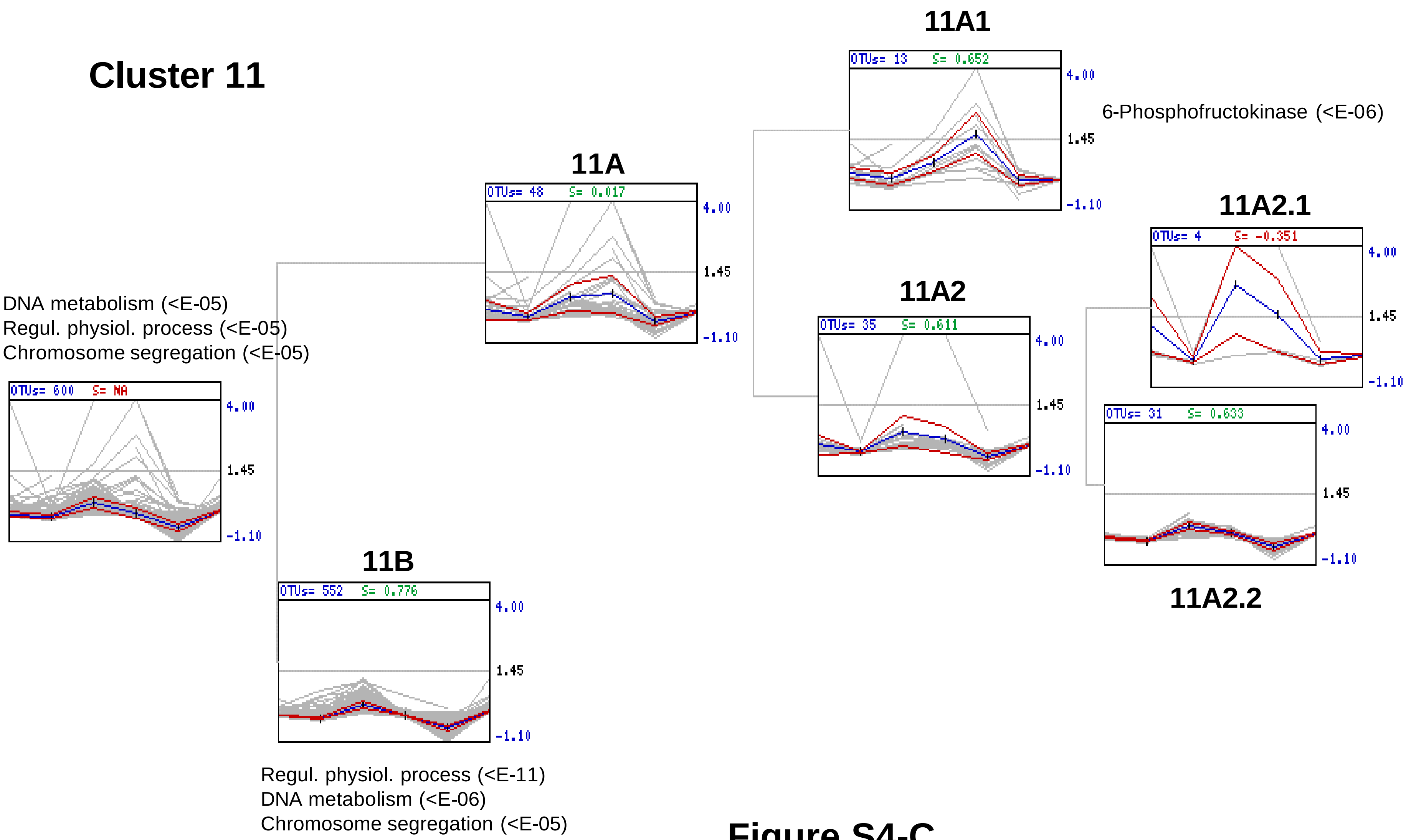
Cluster 11
DNA metabolism (<E-05)
Regul. physiol. process (<E-05)
Chromosome segregation (<E-05)
OTUs= 600 S= NA
4.00
1.45
-1.10
11A
OTUs= 48 S= 0.017
4.00
1.45
-1.10
11B
OTUs= 552 S= 0.776
4.00
1.45
-1.10
Regul. physiol. process (<E-11)
DNA metabolism (<E-06)
Chromosome segregation (<E-05)
11A1
OTUs= 13 S= 0.652
4.00
1.45
-1.10
6-Phosphofructokinase (<E-06)
11A2
OTUs= 35 S= 0.611
4.00
1.45
-1.10
11A2.1
OTUs= 4 S= -0.351
4.00
1.45
-1.10
OTUs= 31 S= 0.633
4.00
1.45
-1.10
11A2.2

Figure S4-C
