# Supplementary material for: Common gene expression strategies revealed by genome-wide analysis in yeast
Source: Genome Biol. 2007 Oct 19;8(10):R222. doi: 10.1186/gb-2007-8-10-r222 (PMC2246296; doi:10.1186/gb-2007-8-10-r222)
Supplement: Additional data file 8 — Summary of numerical data used in the paper. [file gb-2007-8-10-r222-S8.pdf]

Table S1

1. Total number of genes in Table S2 (with at least 1 data point for any variable): **6173**

2. Total number of genes available for each variable, after dubious ORF filtering.

RA	TR	RS	PA	TLRi	TLR	PS
**6117**	**5669**	**4544**	**4239**	**5975**	**5968**	**3367**

3. Total number of genes with at least 1 analysis (with data points for a minimum of 2 variables): **5968**

4. Number of genes considered in each variable pair-wise comparison (Spearman correlation and quintile analyses in Figure 2)

	RA	TR	RS	PA	TLRi	TLR	PS
RA	-						
TR	**5626**	-					
RS	**4537**	**4272**	-				
PA	**4215**	**4020**	**3311**	-			
TLRi	**5968**	**5590**	**4532**	**4192**	-		
TLR	*NA*	**5587**	**4527**	**4189**	*NA*	-	
PS	**3355**	**3207**	**2618**	**3125**	**3343**	**3341**	-

5. Total number of genes with values for at least 5 variables (used as z-scores values for cluster analysis in Figure 3): **3991**

6. Total number of genes with values for the 6 variables: **2338**

7. Number of genes belonging to MIPS complexes containing more than 2 proteins: **2264**. There are available data for **2252** genes.

8. Total number of genes analyzed in Gasch *et al.* [14] with data for fold-change in at least 1 experiment: **6152**. From them we have at least variable data point for **5978** genes.
