# Supplementary material for: Common gene expression strategies revealed by genome-wide analysis in yeast
Source: Genome Biol. 2007 Oct 19;8(10):R222. doi: 10.1186/gb-2007-8-10-r222 (PMC2246296; doi:10.1186/gb-2007-8-10-r222)
Supplement: Additional data file 9 — Ribosome biogenesis genes that appear within the low correlation class in Figure 2b. [file gb-2007-8-10-r222-S9.pdf]

Table S3

ORF	Gene
YDL031W	DBP10
YDL213C	NOP6
YDR449C	UTP6
YER126C	NSA2
YGL078C	DBP3
YGL171W	ROK1
YGR095C	RRP46
YGR103W	NOP7
YGR128C	UTP8
YHR052W	CIC1
YHR197W	RIX1
YLL034C	RIX7
YLR222C	UTP13
YLR276C	DBP9
YMR049C	ERB1
YNL002C	RLP7
YNL061W	NOP2
YOR004W	UTP23
YOR056C	NOB1
YOR206W	NOC2
YOR272W	YTM1
YPL043W	NOP4
YPL266W	DIM1
YPR143W	RRP15
