# Supplementary material for: Common gene expression strategies revealed by genome-wide analysis in yeast
Source: Genome Biol. 2007 Oct 19;8(10):R222. doi: 10.1186/gb-2007-8-10-r222 (PMC2246296; doi:10.1186/gb-2007-8-10-r222)
Supplement: Additional data file 10 — Complete list of significant GO categories found in clusters from Figure 3. [file gb-2007-8-10-r222-S10.pdf]

Table S4

Cluster 2
(385)

Rank	N	X	LOD	P	P-adj	GO Attribute
1	22	54	0.834	1.1e-09	<0.001	0009100: glycoprotein metabolism
2	21	53	0.813	4.7e-09	<0.001	0009101: glycoprotein biosynthesis
3	20	50	0.819	9.1e-09	<0.001	0043413:
4	20	50	0.819	9.1e-09	<0.001	0006486: protein amino acid glycosylation
5	15	33	0.910	8.9e-08	<0.001	0006487: N-linked glycosylation
6	181	1391	0.246	1.3e-07	<0.001	0003824: catalytic activity/enzyme activity
7	53	295	0.350	2.9e-06	0.001	0005783: endoplasmic reticulum/ER
8	20	68	0.615	3.1e-06	0.001	0016757: transferase activity, transferring glycosyl groups/glycosyltransferase
9	16	52	0.641	1.6e-05	0.008	0016758: transferase activity, transferring hexosyl groups/hexosyltransferase
10	12	32	0.768	2e-05	0.009	0000030: mannosyltransferase activity
11	30	152	0.388	8.3e-05	0.037	0044255: cellular lipid metabolism

Cluster 3
(783)

Rank	N	X	LOD	P	P-adj	GO Attribute
1	86	163	0.699	5e-22	<0.001	0007046: ribosome biogenesis
2	93	194	0.617	6.1e-20	<0.001	0007028: cytoplasm organization and biogenesis
3	93	194	0.617	6.1e-20	<0.001	0042254: ribosome biogenesis and assembly
4	69	132	0.682	1.6e-17	<0.001	0006364: rRNA processing
5	69	140	0.630	9e-16	<0.001	0016072: rRNA metabolism
6	83	185	0.557	1.1e-15	<0.001	0005730: nucleolus
7	121	361	0.354	4.3e-11	<0.001	0016070: RNA metabolism
8	30	50	0.799	3.2e-10	<0.001	0006365: 35S primary transcript processing
9	88	255	0.363	5.5e-09	<0.001	0006396: RNA processing
10	13	15	1.351	4.2e-08	<0.001	0042273: ribosomal large subunit biogenesis
11	63	172	0.396	8.1e-08	<0.001	0006519: amino acid and derivative metabolism
12	19	29	0.890	8.3e-08	<0.001	0003743: translation initiation factor activity
13	732	3525	0.321	1.2e-07	<0.001	0005622: intracellular/protoplasm
14	59	160	0.400	1.6e-07	<0.001	0006520: amino acid metabolism
15	22	39	0.731	3.6e-07	<0.001	0006413: translational initiation/protein synthesis initiation
16	64	184	0.361	5.7e-07	0.001	0009308: amine metabolism
17	494	2206	0.173	5.9e-07	0.001	0044237: cellular metabolism
18	176	658	0.215	7e-07	0.001	0043228: non-membrane-bound organelle
19	176	658	0.215	7e-07	0.001	0043232: intracellular non-membrane-bound organelle
20	501	2245	0.172	7.1e-07	0.001	0008152: metabolism/metabolic process
21	647	3042	0.205	1.2e-06	0.002	0050875: cellular physiological process/cell growth and/or maintenance/cell physiology
22	759	3725	0.397	1.4e-06	0.002	0005623: cell
23	66	197	0.336	1.8e-06	0.002	0006807: nitrogen compound metabolism
24	459	2047	0.159	3.1e-06	0.002	0044238: primary metabolism
25	658	3120	0.199	4.5e-06	0.002	0007582: physiological process
26	648	3064	0.194	4.7e-06	0.002	0009987: cellular process
27	21	41	0.641	5.4e-06	0.002	0008135: translation factor activity
28	19	36	0.667	8.7e-06	0.004	0009066: aspartate family amino acid metabolism
29	199	788	0.180	9.2e-06	0.004	0006996: organelle organization and biogenesis
30	8	9	1.369	1.6e-05	0.006	0000176: nuclear exosome (RNase complex)
31	23	51	0.537	2.9e-05	0.010	0045182: translation regulator activity
32	28	69	0.459	4.4e-05	0.016	0009451: RNA modification
33	8	10	1.147	6.6e-05	0.027	0000178: exosome (RNase complex)
34	25	60	0.477	6.7e-05	0.027	0008168: methyltransferase activity/methylase

35	7	8	1.314	7.3e-05	0.032	0000177: cytoplasmic exosome (RNase complex)
36	37	104	0.368	8.6e-05	0.032	0043037: translation/protein translation
37	84	295	0.233	9.2e-05	0.034	0005783: endoplasmic reticulum/ER
38	25	62	0.453	0.00013	0.046	0016741: transferase activity, transferring one-carbon groups
39	35	99	0.363	0.00015	0.054	0044271: nitrogen compound biosynthesis
40	35	99	0.363	0.00015	0.054	0009309: amine biosynthesis
41	70	240	0.245	0.00016	0.060	0019752: carboxylic acid metabolism
42	70	240	0.245	0.00016	0.060	0006082: organic acid metabolism
43	20	46	0.508	0.00018	0.065	0051082: unfolded protein binding
44	19	43	0.520	0.0002	0.070	0000502: proteasome complex (sensu Eukaryota)/26S proteasome

Cluster 4
(136)

Rank	N	X	LOD	P	P-adj	GO Attribute
1	122	2838	0.548	1e-07	<0.001	0043229: intracellular organelle
2	122	2838	0.548	1e-07	<0.001	0043226: organelle
3	60	1020	0.377	1.4e-06	<0.001	0043234: protein complex
4	22	214	0.572	2.2e-06	<0.001	0005840: ribosome
5	9	40	0.956	5.3e-06	<0.001	0015078: hydrogen ion transporter activity/proton transporter
6	9	42	0.929	8.2e-06	<0.001	0015077: monovalent inorganic cation transporter activity/univalent inorganic cation transporter
7	16	133	0.639	8.3e-06	<0.001	0005759: mitochondrial matrix/mitochondrial stroma
8	8	33	0.997	1e-05	0.001	0006119: oxidative phosphorylation/respiratory-chain phosphorylation
9	18	167	0.587	1e-05	0.002	0003735: structural constituent of ribosome/ribosomal protein
10	111	2593	0.382	1.2e-05	0.002	0043231: intracellular membrane-bound organelle
11	111	2593	0.382	1.2e-05	0.002	0043227: membrane-bound organelle
12	27	342	0.449	2.2e-05	0.007	0030529: ribonucleoprotein complex/RNP
13	17	168	0.554	4.3e-05	0.016	0006091: generation of precursor metabolites and energy/energy pathways
14	10	64	0.762	4.8e-05	0.016	0005761: mitochondrial ribosome
15	10	64	0.762	4.8e-05	0.016	0000313: organellar ribosome
16	6	21	1.091	5e-05	0.019	0005746: mitochondrial electron transport chain/respiratory chain
17	12	97	0.646	9.1e-05	0.023	0015934: large ribosomal subunit
18	7	34	0.906	0.00011	0.034	0015399: primary active transporter activity
19	21	256	0.456	0.00012	0.035	0005198: structural molecule activity

Cluster 6
(21)

Rank	N	X	LOD	P	P-adj	GO Attribute
1	12	626	0.856	1.5e-05	0.001	0044249: cellular biosynthesis
2	8	256	0.974	2.5e-05	0.003	0005198: structural molecule activity
3	6	125	1.130	3.1e-05	0.007	0005830: cytosolic ribosome (sensu Eukaryota)/80S ribosome
4	12	692	0.803	4.3e-05	0.011	0009058: biosynthesis/anabolism
5	4	48	1.355	9.5e-05	0.044	0005843: cytosolic small ribosomal subunit (sensu Eukaryota)/40S ribosomal subunit/cytosolic small ribosomal subunit
6	4	48	1.355	9.5e-05	0.044	0016283: eukaryotic 48S initiation complex

Cluster 7
(597)

Rank	N	X	LOD	P	P-adj	GO Attribute
1	105	385	0.376	3.2e-11	<0.001	0006350: transcription
2	112	433	0.345	2.3e-10	<0.001	0051244: regulation of cellular physiological process

3	115	449	0.340	2.4e-10	<0.001	0050789: regulation of biological process/regulation
4	112	434	0.343	2.7e-10	<0.001	0050794: regulation of cellular process
5	114	445	0.340	2.9e-10	<0.001	0050791: regulation of physiological process
6	213	1031	0.242	4.9e-09	<0.001	0006139: nucleobase, nucleoside, nucleotide and nucleic acid metabolism
7	93	357	0.341	7.3e-09	<0.001	0006351: transcription, DNA-dependent
8	57	186	0.428	2.1e-08	<0.001	0000278: mitotic cell cycle
9	74	270	0.364	3.2e-08	<0.001	0019219: regulation of nucleobase, nucleoside, nucleotide and nucleic acid metabolism
10	71	256	0.370	3.7e-08	<0.001	0045449: regulation of transcription
11	74	279	0.344	1.4e-07	<0.001	0007049: cell cycle/cell-division cycle
12	80	310	0.329	1.5e-07	<0.001	0031323: regulation of cellular metabolism
13	68	252	0.352	2.4e-07	<0.001	0030528: transcription regulator activity
14	505	3064	0.249	3e-07	<0.001	0009987: cellular process
15	66	244	0.353	3.3e-07	<0.001	0006355: regulation of transcription, DNA-dependent
16	45	144	0.435	3.7e-07	<0.001	0006357: regulation of transcription from RNA polymerase II promoter/regulation of transcription from Pol II promoter
17	82	328	0.310	4.5e-07	<0.001	0019222: regulation of metabolism
18	60	222	0.350	1.2e-06	0.001	0006366: transcription from RNA polymerase II promoter/transcription from Pol II promoter
19	499	3042	0.228	1.8e-06	0.001	0050875: cellular physiological process/cell growth and/or maintenance/cell physiology
20	36	111	0.456	2.2e-06	0.001	0000074: regulation of cell cycle/cell cycle control
21	48	170	0.372	4.1e-06	0.002	0000279: M phase/M-phase
22	37	119	0.429	4.8e-06	0.002	0000087: M phase of mitotic cell cycle/M-phase of mitotic cell cycle
23	36	117	0.422	8.4e-06	0.005	0007067: mitosis
24	506	3120	0.216	1e-05	0.005	0007582: physiological process
25	94	421	0.244	1.3e-05	0.008	0043412:
26	80	344	0.265	1.3e-05	0.008	0006464: protein modification
27	35	115	0.414	1.5e-05	0.008	0030427: site of polarized growth/site of polarized growth (sensu Fungi)/site of polarized growth (sensu Saccharomyces)
28	17	40	0.635	2.3e-05	0.011	0007088: regulation of mitosis
29	34	114	0.401	3.1e-05	0.016	0005933: bud
30	25	74	0.478	3.7e-05	0.016	0006468: protein amino acid phosphorylation
31	30	97	0.423	4.3e-05	0.017	0004672: protein kinase activity
32	14	31	0.680	5.5e-05	0.021	0005937: mating projection/conjugation tube/shmoo tip
33	14	31	0.680	5.5e-05	0.021	0042995: cell projection
34	62	263	0.267	9.1e-05	0.046	0007275: development
35	12	25	0.728	9.1e-05	0.047	0043332: mating projection tip/conjugation tube tip
36	20	56	0.514	9.1e-05	0.048	0004674: protein serine/threonine kinase activity
37	258	1447	0.149	9.3e-05	0.050	0005634: nucleus

Cluster 8
(96)

Rank	N	X	LOD	P	P-adj	GO Attribute
1	72	125	2.327	9e-98	<0.001	0005830: cytosolic ribosome (sensu Eukaryota)/80S ribosome
2	72	167	2.071	2.3e-85	<0.001	0003735: structural constituent of ribosome/ribosomal protein
3	74	214	1.947	1.4e-79	<0.001	0005840: ribosome
4	72	256	1.774	3.5e-69	<0.001	0005198: structural molecule activity
5	73	299	1.704	2.2e-65	<0.001	0005829: cytosol
6	75	342	1.677	5.6e-64	<0.001	0030529: ribonucleoprotein complex/RNP
7	76	377	1.648	3.8e-62	<0.001	0006412: protein biosynthesis
8	43	62	2.208	1.4e-59	<0.001	0005842: cytosolic large ribosomal subunit (sensu Eukaryota)/60S ribosomal subunit/cytosolic large ribosomal subunit
9	76	414	1.593	8.4e-59	<0.001	0009059: macromolecule biosynthesis

10	43	97	1.758	1.5e-47	<0.001	0015934: large ribosomal subunit
11	78	626	1.413	2.5e-47	<0.001	0044249: cellular biosynthesis
12	78	692	1.355	7.3e-44	<0.001	0009058: biosynthesis/anabolism
13	74	658	1.273	2.8e-40	<0.001	0043228: non-membrane-bound organelle
14	74	658	1.273	2.8e-40	<0.001	0043232: intracellular non-membrane-bound organelle
15	29	48	1.938	9.7e-37	<0.001	0005843: cytosolic small ribosomal subunit (sensu Eukaryota)/40S ribosomal subunit/cytosolic small ribosomal subunit
16	29	48	1.938	9.7e-37	<0.001	0016283: eukaryotic 48S initiation complex
17	77	892	1.176	4.2e-34	<0.001	0019538: protein metabolism/protein metabolism and modification
18	76	874	1.160	1.4e-33	<0.001	0044267: cellular protein metabolism
19	29	58	1.758	2.1e-33	<0.001	0016282: eukaryotic 43S preinitiation complex/eukaryotic 43S pre-initiation complex
20	80	1020	1.185	3.4e-33	<0.001	0043234: protein complex
21	77	988	1.114	8.6e-31	<0.001	0044260: cellular macromolecule metabolism
22	29	74	1.568	1.7e-29	<0.001	0015935: small ribosomal subunit
23	78	1065	1.096	1.8e-29	<0.001	0043170: macromolecule metabolism
24	83	2047	0.783	2.2e-13	<0.001	0044238: primary metabolism
25	85	2245	0.775	4.5e-12	<0.001	0008152: metabolism/metabolic process
26	84	2206	0.751	7.5e-12	<0.001	0044237: cellular metabolism
27	88	2594	0.760	9.1e-10	<0.001	0005737: cytoplasm
28	11	41	1.231	1.5e-09	<0.001	0042257: ribosomal subunit assembly
29	11	50	1.118	1.5e-08	<0.001	0042255: ribosome assembly
30	6	11	1.705	7e-08	<0.001	0000028: ribosomal small subunit assembly and maintenance
31	92	3042	0.817	1.5e-07	<0.001	0050875: cellular physiological process/cell growth and/or maintenance/cell physiology
32	92	3064	0.803	2.6e-07	<0.001	0009987: cellular process
33	92	3120	0.768	1.1e-06	0.001	0007582: physiological process
34	87	2838	0.582	2.7e-06	0.001	0043229: intracellular organelle
35	87	2838	0.582	2.7e-06	0.001	0043226: organelle
36	4	6	1.879	4.6e-06	0.002	0006450: regulation of translational fidelity/regulation of translational accuracy
37	11	108	0.719	4.4e-05	0.015	0006461: protein complex assembly/chaperone activity
38	15	194	0.595	4.7e-05	0.016	0007028: cytoplasm organization and biogenesis
39	15	194	0.595	4.7e-05	0.016	0042254: ribosome biogenesis and assembly

Cluster 9
(72)

Rank	N	X	LOD	P	P-adj	GO Attribute
1	39	1250	0.420	4.3e-05	0.015	0005554: molecular function unknown

Cluster 11
(600)

Rank	N	X	LOD	P	P-adj	GO Attribute
1	77	310	0.301	1.7e-06	0.001	0031323: regulation of cellular metabolism
2	78	328	0.275	8.8e-06	0.003	0019222: regulation of metabolism
3	80	342	0.265	1.3e-05	0.005	0006259: DNA metabolism
4	66	270	0.287	1.8e-05	0.007	0019219: regulation of nucleobase, nucleoside, nucleotide and nucleic acid metabolism
5	95	433	0.231	3.2e-05	0.009	0051244: regulation of cellular physiological process
6	95	434	0.230	3.5e-05	0.011	0050794: regulation of cellular process
7	96	445	0.221	5.8e-05	0.016	0050791: regulation of physiological process
8	26	80	0.450	6e-05	0.016	0007059: chromosome segregation/chromosome transmission
9	17	43	0.580	7.6e-05	0.030	0005083: small GTPase regulator activity/small GTPase regulatory/interacting protein activity
10	96	449	0.215	8.4e-05	0.045	0050789: regulation of biological process/regulation

Cluster 20
(103)

Rank	N	X	LOD	P	P-adj	GO Attribute
1	3	4	1.955	6.6e-05	0.021	0004180: carboxypeptidase activity

Cluster 25
(3)

Rank	N	X	LOD	P	P-adj	GO Attribute
1	2	48	2.150	0.00042	0.038	0006006: glucose metabolism
2	2	61	2.041	0.00068	0.048	0019318: hexose metabolism

Rank: position in the attribute list ranked by significance of association with your query;
N: number of genes in your query with this attribute;
X: number of genes overall with this attribute;
LOD: the logarithm (base 10) of the odds ratio; positive and negative values indicate over- and under-representation, respectively;
P: single hypothesis one-sided P-value of the association between attribute and query (based on Fisher's Exact Test);
P-adj: adjusted P-value: fraction (as a %) of 1000 null-hypothesis simulations having attributes with this single-hypothesis P value or smaller
