# Supplementary material for: Common gene expression strategies revealed by genome-wide analysis in yeast
Source: Genome Biol. 2007 Oct 19;8(10):R222. doi: 10.1186/gb-2007-8-10-r222 (PMC2246296; doi:10.1186/gb-2007-8-10-r222)
Supplement: Additional data file 11 — Complete list of significant GO categories found in clusters from Additional data files 2 and 3 and not present in Figure 3 [file gb-2007-8-10-r222-S11.pdf]

Table S5

Figure S2 (20 clusters)

Cluster 9 + 10
(406)

Rank	N	X	LOD	P	P-adj	GO Attribute
1	163	1250	0.188	4.7e-05	0.017	0005554: molecular function unknown

Cluster 12 + 13 + 14
(400)

Rank	N	X	LOD	P	P-adj	GO Attribute
1	5	6	1.522	5.5e-05	0.020	0005984: disaccharide metabolism

Cluster 17 + 18
(14)

No significant GO categories were found.

Cluster 24 + 25
(7)

No significant GO categories were found.

Figure S3 (25 clusters)

a) Included in Cluster 3 from Figure 3
(601)

Rank	N	X	LOD	P	P-adj	GO Attribute
1	63	163	0.586	4.4e-14	<0.001	0007046: ribosome biogenesis
2	68	194	0.520	1.1e-12	<0.001	0007028: cytoplasm organization and biogenesis
3	68	194	0.520	1.1e-12	<0.001	0042254: ribosome biogenesis and assembly
4	52	132	0.594	3.6e-12	<0.001	0006364: rRNA processing
5	52	140	0.552	5.1e-11	<0.001	0016072: rRNA metabolism
6	62	185	0.486	1e-10	<0.001	0005730: nucleolus
7	68	255	0.341	5e-07	<0.001	0006396: RNA processing
8	22	50	0.660	7.9e-07	0.001	0006365: 35S primary transcript processing
9	87	361	0.285	1.5e-06	0.001	0016070: RNA metabolism
10	8	9	1.510	2e-06	0.002	0000176: nuclear exosome (RNase complex)
11	160	788	0.203	5.5e-06	0.003	0006996: organelle organization and biogenesis
12	10	15	1.038	8.3e-06	0.003	0042273: ribosomal large subunit biogenesis
13	8	10	1.288	8.7e-06	0.003	0000178: exosome (RNase complex)
14	561	3525	0.296	9.8e-06	0.004	0005622: intracellular/protoplasm
15	7	8	1.454	1.2e-05	0.004	0000177: cytoplasmic exosome (RNase complex)
16	135	658	0.201	2.3e-05	0.005	0043228: non-membrane-bound organelle
17	135	658	0.201	2.3e-05	0.005	0043232: intracellular non-membrane-bound organelle
18	9	14	0.994	3.7e-05	0.010	0000175: 3'-5'-exoribonuclease activity/3'-5' exoribonuclease activity
19	172	886	0.177	3.9e-05	0.011	0016043: cell organization and biogenesis
20	18	46	0.572	5.6e-05	0.020	0051082: unfolded protein binding
21	582	3725	0.364	5.7e-05	0.020	0005623: cell

b) Included in Cluster 3 from Figure 3
(133)

Rank	*N*	X	LOD	P	P-adj	GO Attribute
1	21	163	0.697	5.5e-08	<0.001	0007046: ribosome biogenesis
2	22	194	0.634	2.6e-07	<0.001	0007028: cytoplasm organization and biogenesis
3	22	194	0.634	2.6e-07	<0.001	0042254: ribosome biogenesis and assembly
4	15	104	0.741	1.2e-06	<0.001	0043037: translation/protein translation
5	15	132	0.619	2.5e-05	0.008	0006364: rRNA processing
6	18	185	0.547	3.2e-05	0.009	0005730: nucleolus
7	7	29	1.004	3.3e-05	0.009	0003743: translation initiation factor activity
8	27	361	0.434	3.9e-05	0.014	0016070: RNA metabolism
9	8	41	0.888	4.7e-05	0.019	0008135: translation factor activity, nucleic acid binding
10	15	140	0.590	5e-05	0.019	0016072: rRNA metabolism
11	16	160	0.557	6.5e-05	0.023	0006520: amino acid metabolism
12	18	197	0.516	7.3e-05	0.027	0006807: nitrogen compound metabolism
13	10	69	0.734	7.7e-05	0.031	0009451: RNA modification

c) Included in Cluster 3 from Figure 3
(49)

Rank	N	X	LOD	P	P-adj	GO Attribute
1	18	240	0.992	1.2e-10	<0.001	0019752: carboxylic acid metabolism
2	18	240	0.992	1.2e-10	<0.001	0006082: organic acid metabolism
3	13	160	0.978	3.2e-08	<0.001	0006520: amino acid metabolism
4	13	172	0.943	7.7e-08	<0.001	0006519: amino acid and derivative metabolism
5	13	184	0.910	1.7e-07	<0.001	0009308: amine metabolism
6	13	197	0.877	3.8e-07	<0.001	0006807: nitrogen compound metabolism
7	6	36	1.282	3.7e-06	0.002	0009066: aspartate family amino acid metabolism
8	40	2047	0.613	9.5e-06	0.008	0044238: primary metabolism
9	21	692	0.565	2.4e-05	0.012	0009058: biosynthesis/anabolism
10	4	15	1.529	2.5e-05	0.013	0009081: branched chain family amino acid metabolism
11	44	2594	0.644	6.9e-05	0.024	0005737: cytoplasm
12	48	3120	0.960	8.3e-05	0.025	0007582: physiological process
13	40	2206	0.543	9.4e-05	0.028	0044237: cellular metabolism

d) Included in Cluster 8 from Figure 3
(15)

Rank	N	X	LOD	P	P-adj	GO Attribute
1	8	125	1.570	4e-09	<0.001	0005830: cytosolic ribosome (sensu Eukaryota)/80S ribosome
2	8	167	1.433	4e-08	<0.001	0003735: structural constituent of ribosome/ribosomal protein
3	6	62	1.676	5e-08	<0.001	0005842: cytosolic large ribosomal subunit (sensu Eukaryota)/60S ribosomal subunit/cytosolic large ribosomal subunit
4	8	214	1.315	2.8e-07	<0.001	0005840: ribosome
5	9	342	1.203	7.1e-07	<0.001	0030529: ribonucleoprotein complex/RNP
6	6	97	1.463	7.4e-07	<0.001	0015934: large ribosomal subunit
7	11	626	1.144	9.8e-07	<0.001	0044249: cellular biosynthesis
8	8	256	1.230	1.1e-06	<0.001	0005198: structural molecule activity
9	9	377	1.155	1.6e-06	<0.001	0006412: protein biosynthesis
10	11	692	1.091	2.8e-06	<0.001	0009058: biosynthesis/anabolism
11	9	414	1.109	3.7e-06	<0.001	0009059: macromolecule biosynthesis
12	8	299	1.156	3.7e-06	<0.001	0005829: cytosol
13	12	1020	1.021	1.5e-05	0.009	0043234: protein complex

e) Included in Cluster 8 from Figure 3
(16)

Rank	N	X	LOD	P	P-adj	GO Attribute
1	13	214	1.858	1e-14	<0.001	0005840: ribosome
2	11	125	1.848	7.1e-14	<0.001	0005830: cytosolic ribosome (sensu Eukaryota)/80S ribosome
3	11	167	1.707	1.8e-12	<0.001	0003735: structural constituent of ribosome/ribosomal protein
4	13	342	1.630	4.9e-12	<0.001	0030529: ribonucleoprotein complex/RNP
5	13	377	1.582	1.7e-11	<0.001	0006412: protein biosynthesis
6	13	414	1.535	5.7e-11	<0.001	0009059: macromolecule biosynthesis
7	11	256	1.502	2e-10	<0.001	0005198: structural molecule activity
8	11	299	1.426	1.1e-09	<0.001	0005829: cytosol
9	13	626	1.325	1.1e-08	<0.001	0044249: cellular biosynthesis
10	6	48	1.758	1.6e-08	<0.001	0005843: cytosolic small ribosomal subunit (sensu Eukaryota)/40S ribosomal subunit/cytosolic small ribosomal subunit
11	6	48	1.758	1.6e-08	<0.001	0016283: eukaryotic 48S initiation complex
12	13	658	1.298	2.1e-08	<0.001	0043228: non-membrane-bound organelle
13	13	658	1.298	2.1e-08	<0.001	0043232: intracellular non-membrane-bound organelle
14	13	692	1.272	3.9e-08	<0.001	0009058: biosynthesis/anabolism
15	6	58	1.665	5.2e-08	<0.001	0016282: eukaryotic 43S preinitiation complex/eukaryotic 43S pre-initiation complex
16	6	74	1.547	2.3e-07	<0.001	0015935: small ribosomal subunit
17	13	874	1.144	7.2e-07	<0.001	0044267: cellular protein metabolism
18	13	892	1.132	9.2e-07	<0.001	0019538: protein metabolism/protein metabolism and modification
19	5	62	1.513	3e-06	<0.001	0005842: cytosolic large ribosomal subunit (sensu Eukaryota)/60S ribosomal subunit/cytosolic large ribosomal subunit
20	13	988	1.074	3.2e-06	<0.001	0044260: cellular macromolecule metabolism
21	13	1020	1.055	4.8e-06	<0.001	0043234: protein complex
22	13	1065	1.029	8e-06	<0.001	0043170: macromolecule metabolism
23	5	97	1.302	2.7e-05	0.008	0015934: large ribosomal subunit

f) Included in Cluster 8 from Figure 3
(65)

Rank	N	X	LOD	P	P-adj	GO Attribute
1	53	125	2.357	2.1e-73	<0.001	0005830: cytosolic ribosome (sensu Eukaryota)/80S ribosome
2	53	167	2.153	3.5e-65	<0.001	0003735: structural constituent of ribosome/ribosomal protein
3	53	214	1.999	1.4e-58	<0.001	0005840: ribosome
4	53	256	1.893	5.9e-54	<0.001	0005198: structural molecule activity
5	54	299	1.851	7e-52	<0.001	0005829: cytosol
6	53	342	1.730	9.7e-47	<0.001	0030529: ribonucleoprotein complex/RNP
7	54	377	1.722	4.6e-46	<0.001	0006412: protein biosynthesis
8	32	62	2.093	2.3e-44	<0.001	0005842: cytosolic large ribosomal subunit (sensu Eukaryota)/60S ribosomal subunit/cytosolic large ribosomal subunit
9	54	414	1.671	9.4e-44	<0.001	0009059: macromolecule biosynthesis
10	32	97	1.757	1.7e-36	<0.001	0015934: large ribosomal subunit
11	54	626	1.443	8.5e-34	<0.001	0044249: cellular biosynthesis
12	54	692	1.387	1.9e-31	<0.001	0009058: biosynthesis/anabolism
13	53	658	1.370	3.2e-31	<0.001	0043228: non-membrane-bound organelle
14	53	658	1.370	3.2e-31	<0.001	0043232: intracellular non-membrane-bound organelle
15	21	48	1.835	1.3e-26	<0.001	0005843: cytosolic small ribosomal subunit (sensu Eukaryota)/40S ribosomal subunit/cytosolic small ribosomal subunit
16	21	48	1.835	1.3e-26	<0.001	0016283: eukaryotic 48S initiation complex

17	54	874	1.253	4.9e-26	<0.001	0044267: cellular protein metabolism
18	54	892	1.241	1.4e-25	<0.001	0019538: protein metabolism/protein metabolism and modification
19	21	58	1.699	1.8e-24	<0.001	0016282: eukaryotic 43S preinitiation complex/eukaryotic 43S pre-initiation complex
20	55	988	1.229	1.9e-24	<0.001	0044260: cellular macromolecule metabolism
21	55	1020	1.209	1e-23	<0.001	0043234: protein complex
22	55	1065	1.183	1e-22	<0.001	0043170: macromolecule metabolism
23	21	74	1.543	6.8e-22	<0.001	0015935: small ribosomal subunit
24	10	41	1.369	4.9e-10	<0.001	0042257: ribosomal subunit assembly
25	10	50	1.259	4e-09	<0.001	0042255: ribosome assembly
26	6	11	1.891	6.4e-09	<0.001	0000028: ribosomal small subunit assembly and maintenance
27	55	2047	0.709	1.5e-08	<0.001	0044238: primary metabolism
28	56	2206	0.690	9.2e-08	<0.001	0044237: cellular metabolism
29	56	2245	0.673	2.1e-07	<0.001	0008152: metabolism/metabolic process
30	60	2594	0.780	2.7e-07	<0.001	0005737: cytoplasm
31	4	6	2.060	9.4e-07	0.001	0006450: regulation of translational fidelity/regulation of translational accuracy
32	14	194	0.766	1.8e-06	0.001	0007028: cytoplasm organization and biogenesis
33	14	194	0.766	1.8e-06	0.001	0042254: ribosome biogenesis and assembly
34	10	108	0.866	7.2e-06	0.002	0006461: protein complex assembly/chaperone activity
35	62	3042	0.752	3.2e-05	0.014	0050875: cellular physiological process/cell growth and/or maintenance/cell physiology
36	62	3064	0.738	4.7e-05	0.020	0009987: cellular process
37	5	26	1.217	5e-05	0.020	0006417: regulation of protein biosynthesis
38	5	26	1.217	5e-05	0.020	0006445: regulation of translation
39	59	2838	0.576	0.00011	0.039	0043229: intracellular organelle
40	59	2838	0.576	0.00011	0.039	0043226: organelle
41	62	3120	0.703	0.00012	0.040	0007582: physiological process

g) Included in Cluster 9 from Figure 3
(24)

No significant GO categories were found.

h) Included in Cluster 9 from Figure 3
(48)

No significant GO categories were found.
