# Supplementary material for: Common gene expression strategies revealed by genome-wide analysis in yeast
Source: Genome Biol. 2007 Oct 19;8(10):R222. doi: 10.1186/gb-2007-8-10-r222 (PMC2246296; doi:10.1186/gb-2007-8-10-r222)
Supplement: Additional data file 12 — Standard error averages calculated for experimental (aSEe) and random sampling (aSEr) estimations for the functionally related groups from Figure 4 [file gb-2007-8-10-r222-S12.pdf]

Table S6

Group	Sub-group	n	aSEe	aSEr
**Nucleosome**		8	0.0242	0.1019
**TOM-TIM**		16	0.0604	0.0793
**Respiratory complexes**	**COX**	9	0.0865	0.0987
	**Cit b/c**	9	0.0724	0.0904
	**ATP synthase**	17	0.0478	0.0763
**Cytosolic ribosome**		137	0.0102	0.0249
**Proteasome**	**20S**	14	0.0313	0.0820
	**19S**	19	0.0456	0.0653
**RNA polymerases**		23	0.0473	0.0626
**SAGA**		17	0.0608	0.0694
**90S Processosome**		52	0.0281	0.0404
**Nuclear pore**		48	0.0356	0.0416
**Mitochondrial ribosome**		67	0.0271	0.0362
**Exosome**		14	0.0475	0.0782
**Spliceosome**		30	0.0403	0.0573
**APC**		16	0.0729	0.0773
**Energy pathways**	**Glycolysis & Gluconeogenesis**	41	0.0479	0.0458
	**TCA**	31	0.0506	0.0535
	**Fermentation**	33	0.0576	0.0562
**Vacuole**		18	0.0614	0.0707
**Transcription factors**		145	0.0225	0.0247
**Replication complexes**		175	0.0359	0.0416
**Mitosis**		145	0.0237	0.0241
