# Supplementary material for: Common gene expression strategies revealed by genome-wide analysis in yeast
Source: Genome Biol. 2007 Oct 19;8(10):R222. doi: 10.1186/gb-2007-8-10-r222 (PMC2246296; doi:10.1186/gb-2007-8-10-r222)
Supplement: Additional data file 14 — Description and comments of the data shown in additional data file 1. [file gb-2007-8-10-r222-S14.pdf]

Figure S1. Plot of abundance and stability for mRNA and protein molecules. Abundance (in molecules/cell) and stability (in minutes) values for 2338 genes whose mRNA and protein are shown as black and grey dots, respectively. Units are given in a logarithmic scale because of the range dispersion between both kinds of molecules. Cytosolic ribosomal protein genes (dark and light blue dots for mRNA and protein, respectively) and mitochondrial ribosomal protein genes (green and light green dots for mRNA and protein, respectively) are highlighted over the global distribution. Red dots represent the average value for cytosolic protein genes that are rather different from the whole set of genes analyzed (orange dots) whereas the behavior of mitochondrial ribosome genes (yellow dots) is very similar to the whole.

It can be seen that mRNAs and proteins spanned a range of about 3.5 orders when the least and the most abundant molecules were compared. Proteins were about 3 orders of magnitude more abundant. On the contrary, mRNA half-lives were concentrated in a 1.5 orders-wide range while protein stabilities displayed a range of, again, about 3 orders of magnitude, due to the existence of some long-lived proteins. It is interesting to note that some well-defined (structurally or functionally) groups of genes occupied restricted areas in both distributions. In the Figure is shown that 60 cytosolic ribosomal protein genes analyzed (blue diamonds, red dot) indicates that this group was rather different than the whole of the 2340 genes analyzed, but that the 22 mitochondrial ribosomal protein genes (green diamonds, yellow dot) were very close to the average (orange dot).. This is a first indication that functionally related genes tend to have similar parameter levels for mRNA and protein.

Figure S2. SOTA dendrogram obtained by using a variability threshold yielding 20 clusters. Note that some new clusters are originated by merging some ones from Figure 3 as indicated by the numbers in the right margin of the clusters.

Figure S3. SOTA dendrogram obtained by using a variability threshold yielding 30 clusters. Note that some new clusters are originated by splitting some ones from Figure 3 as indicated by the numbers in the right margin of the clusters.

Figure S4. Further analysis of clusters 3 (A), 7 (B) and 11 (C) using CAAT. The largest clusters (3, 7 and 11) from Figure 4 were split into new clusters using the CAAT program. Significant GO categories in each cluster are marked and the P-values indicated. OTUs (in blue) indicates the number of genes includes in each cluster subdivision. S (in green) represents the Silouette value for each splitting step. The grey lines in each cluster graph correspond to individual gene profiles

Figure S5. Average 6VP for some additional functional groups. See Fig. 4 for details.

Table S1. Number of genes used for each kind of analysis.

Table S2. List of values for the six variables of the 6173 genes analyzed. It contains several sheets shown the different kind of data used in the different analyses.

Table S3. List of genes from the ribosome biogenesis GO category that appear in the low correlation class of Fig. 2B.

Table S4. Complete Gene Ontology (GO) searches for the clusters in Figure 3 by using FuncAssociate web server (only clusters including significant GOs are shown). Numbers in parenthesis below the cluster names indicate cluster sizes**.**

Table S5. List of GO categories found in clusters of Figures S2 and S3. Only GO terms belonging to clusters that did not appear previously in Figure 3 are shown. Numbers in parenthesis below the cluster names indicate cluster sizes. Legends for table headings as in Table S4.

Table S6. Standard Error (SE) averages calculated for experimentally (aSEe) and random sampling (aSEr) estimations for the functionally related groups for which 6VP are shown in Figure 4. n, indicates the group sizes. Red values indicate a higher average error. It can be seen that, for most cases, the average error is higher for the random samples.
